# Enhancement of AVR system performance by using hybrid harmony search and dwarf mongoose optimization algorithms

**DOI:** 10.1038/s41598-024-77120-3

**Published:** 2024-11-08

**Authors:** Omar M. Hesham, Mahmoud A. Attia, S. F. Mekhamer

**Affiliations:** 1 Department of Electrical Power and Machines, Faculty of Engineering, Ain Shams University, Cairo, Egypt; 2 Electrical Engineering Department, Future University in Egypt, New Cairo, Egypt

**Keywords:** AVR, DMOA, HS, PID, PIDA, Hybrid optimizations, Electrical and electronic engineering, Energy infrastructure

## Abstract

Innovations in control algorithms, integration of smart grid technologies, and advancements in materials and manufacturing techniques all push the boundaries of AVR performance. As the demand for power systems progresses with the complexity and variety of loads, conventional AVR designs may struggle to handle these ever-changing circumstances efficiently. Therefore, the need for new optimization methods is crucial to bolstering the efficiency, reliability, and adaptability of AVRs. Thus, this work aims to improve the performance of the AVR system controller by using a novel hybrid technique between the Harmony Search (HS) and Dwarf Mongoose Optimization (DMO) algorithms to tune the proportional-integral-derivative (PID) and proportional-integral-derivative acceleration (PIDA) parameters. The suggested hybrid approach ensures an accurate solution with balanced exploration and exploitation rates. The reliability of the proposed HS-DMOA is verified through comparison with different optimization techniques carried out on time and frequency performance indicators, disturbances in the form of changes to time constants, and dynamic input signals. The proposed hybrid HS-DMOA PID-based has better overshoot than PID-based HS, LUS, TLBO, SMA, RSA, and L-RSAM by 20.37%, 18.5%, 18.5%, 2.77%, 5.55%, and 2.77%, respectively. Regarding the phase margin, the proposed hybrid HS-DMOA PID-based is better than PID-based HS, LUS, and TLBO by 39%, 37%, and 38%, respectively. While the proposed hybrid HS-DMOA PIDA-based has a better overshoot than PIDA-based HS, LUS, and PID HS-DMOA-based by 14%, 17%, and 20%, respectively. Moreover, the robustness under dynamic disturbance proved the reliability of the proposed HS-DMOA PID and PIDA based through enhancement of overshoot around 0.3%~20% for different cases. Finally, the main contribution of the paper is to propose a relatively new hybrid optimization method to enhance the AVR PID and PIDA-based performance with detailed analysis in time and frequency domains under normal and dynamic disturbances.

## Introduction

Voltage fluctuations pose serious challenges in the modern world. Leaning more toward sustainable sources, several countries and researchers worldwide are adopting strict policies urging the transformation from fossil fuel-based power generation methods to other renewable alternatives like solar and wind due to their deficiency in power plants and pollution due to carbon emissions^1,2^. However, the constant loading of systems and renewable energy components, like wind and solar farms, into conventional power grids has provoked electrical complexities^3^. Voltage variation and instability in the power grid might accumulate and bear catastrophic consequences for the electrical network. For instance, in March of 2019, due to overloading an already strained electrical grid, insufficient maintenance of the voltage regulation systems, and poor protection control, Venezuela underwent two successive national blackouts, involving more than 90% of the administrative regions, including the capital Caracas, with almost 30 million people affected^4^. Thus, it has become more crucial, now than ever, to develop a way to constantly stabilize voltage levels across different nodes throughout the network to limit voltage fluctuations.

An Automatic Voltage Regulator (AVR) is a device that calibrates the output voltage of a synchronous generator or an alternator, regardless of fluctuations in load or changes in operating conditions^5^. At first, the AVR senses the output voltage using a sensor, usually a voltage transformer circuit. This measured voltage is then compared to a reference voltage, which refers to the preferred output level. Any difference between the sensed and reference voltages generates an error signal, which is amplified. This amplified error signal controls the electrical current, called the excitation current, passing through the generator’s field winding. The field winding in the rotor generates a magnetic field. This magnetic field interacts with the stator windings, producing induced voltage on the stator’s side. The induced voltage in the stator windings ultimately becomes the output voltage of the generator. However, the AVR system alone may struggle to respond effectively to fluctuations in load demand, input voltage variations, or other external disturbances. Without real-time feedback and dynamic adjustment capabilities, the system may experience voltage deviations, instability, or inefficient operation. Additionally, manual intervention may be required to recalibrate the AVR system or adjust its settings, leading to delays and potential disruptions in power supply.

By leveraging feedback control, dynamic adjustment, remote monitoring, and advanced diagnostics, a controller empowers the AVR system to regulate voltage more effectively, adapt to changing conditions, and ensure a stable and reliable power supply. Two of the most widely used controllers in the literature are the proportional-integral-derivative (PID) and the proportional-integral-derivative-acceleration (PIDA) controllers, mainly due to their design and computational simplicity. A controller typically improves the response and stability of an AVR system by minimizing performance indices, like the maximum percentage overshoot (*Ess*), settling time (*T*_*s*_), rise time (*T*_*r*_), and steady-state error (*M*_*p*_)^6^. Design parameters include factors like the order, time delays, nonlinear loads, and changeable operating points of a system^7^. Modern AVRs, with the aid of sophisticated controllers, can possess exquisite features like protection mechanisms and communication interfaces for long-distance monitoring and controlling, boosting the system’s reliability and performance^8^. At heart, an AVR controller uses various optimization methods designed to monitor the output voltage. The two main general methods for optimization methods are the metaheuristic and mathematical approaches^9^ and ^10^. However, due to the sensitivity of the latter to initial conditions and its gradient-dependent nature, in addition to the increasing complexity of modern-day optimization problems, nature-based algorithms have proven over time their reliable precision and versatile application^11–15^. The main goal of an optimization algorithm is to limit the error between the measured and desired voltages; in other words, to minimize the objective function^16^. An objective function acts as the guiding principle for the controller’s activity, considering factors like the magnitude and duration of voltage fluctuations, as well as any constraints, limitations, or non-linearities imposed on the system. These objective functions are usually utilized to minimize qualitative quantities that help fine-tune the controller parameters, such as the integral of square error (ISE), the integral of absolute error (IAE), and the integral of time-weighted square error (ITSE)^6^.

The optimization of various algorithms in tuning controllers had been the subject of extensive research extending back to the early 2000s. In^17^, it was first claimed that the proposed Particle Swarm Optimization (PSO) method could avoid the shortcomings of premature convergence of the Genetic Algorithm (GA) method while also maintaining a higher quality of solution with better computation efficiency. Thus, the authors concluded that PSO had more robust stability and efficiency and can solve the searching and tuning problems of PID controller parameters more easily and quickly than the GA method. After proposing the original method, typically divergent versions start emerging in hopes of producing more diverse parameters to lead to favorable system characteristics and overcome the original technique’s shortcomings. In the case of the PSO, the algorithm might converge prematurely if particles (individual solution candidates) cluster around local solutions and fail to explore other potential areas of the search space. To address these limitations, the Chaotic Particle Swarm Optimization (CPSO) variant integrates chaotic sequences within the PSO framework to enhance its search technique^17^. Chaotic maps, in this case the logistic map, were employed to introduce controlled randomness into the algorithm, which in turn helped in diversifying the search process, limiting particles from getting stuck in local optima. Another variation of the PSO was the Quantum Gaussian Particle Swarm Optimization (QGPSO). In conventional PSO, each particle had deterministic positions and velocities that guide its motion through the search space, whereas in QGUPSO, they were probabilistic^18^. The quantum mechanics component allows particles to exist in a superposition of states, providing them with the ability to “tunnel” through potential barriers, effectively jumping out of local optima that would trap classical particles. The Gaussian distribution element further enhanced the search process by using a Gaussian distribution centered around the global best solution^18^. Later, the idea of applying optimization algorithms to other variations of the PID controller seemed appealing to the scientific community, however, it didn’t yield quite the anticipated results^19^. In^20^, the PSO algorithm was applied to the fractional order PID (FOPID) controller. While the FOPID-based system showcased a more robust and stable behavior by displaying higher gain and phase margins and a slightly reduced overshoot, it still lagged its PID-based counterpart in terms of settling and rise time. Another approach involved the implementation of the PSO-BELBIC controller, the fitness values of the two controllers were close together, where the PSO-BELBIC controller reduced the percent overshoot of the system more effectively but gave a slower response compared with the standard PSO-PID controller since it had learning capability^21^. Throughout the last decade, PSO had become arguably the most commonly used modern optimization algorithm due to the ease of its implementation in linear or nonlinear continuous numerical optimization problems, in addition to its wide variability to minor and major modifications^22^. Table 1 summarizes a comparative study of different optimization techniques.

**Table 1:** Comparative study between different optimization techniques.

Source	Optimization method	Controller type used	Key takeaways		Year published
			Advantage	Disadvantage	
^23^	Continuous Firefly Algorithm (CFA)	PID	The CFA effectively optimizes the controller parameters in complex systems, outperforming traditional methods like Particle Swarm Optimization in time domain specifications and set-point tracking.	The CFA is sensitive to parameter selection and may require significant computational resources, potentially leading to longer processing times and risks of getting trapped in local optima.	2014
^24^	Jaya optimization algorithm (JOA)	FOPID	59.82%, 56.09%, 14.94%, 34.24%, 35.70%, 21.64%, 12.0%, 41.33%, 14.84% and 15.17% reduced overshoot than that of differential evolution (DE), particle swarm optimization (PSO), Artificial Bee, Colony (ABC), Bibliography Based Optimization (BBO), Grasshopper Optimization Algorithm (GOA), Pattern Search Algorithm (PSA), Improved Kidney Inspired Algorithm (IKA), Whale Optimization Algorithm (WOA), Salp Swarm Algorithm (SSA) and Local Unimodal Sampling (LUS) algorithm respectively	The JOA, while simple and parameter-free, may struggle with premature convergence and local optima in complex problems compared to more sophisticated algorithms	2020
^24^	Water Cycle Algorithm (WCA)	2DOF-PI	WCA has shown superior convergence abilities and provides better performance metrics, such as reduced maximum overshoot and settling time while being simple to implement and requires fewer parameters to tune compared to other algorithms.	The performance of WCA can be affected by the choice of parameters, which may require careful tuning. It may not perform as well in highly dynamic or non-linear systems compared to more complex algorithms	2021
^25^	Sine Cosine Algorithm (SCA)	FOPID	SCA excels in avoiding local minima and improves convergence in complex optimization tasks, outperforming algorithms like PSO, Genetic Algorithm (GA), and Bat Algorithm (BA). It enhances FOPID controllers with better transient responses, lower overshoot, and faster settling time, statistically reducing mean error margins.	SCA may increase computation times due to its complex iterative process, impacting real-time application efficiency. The algorithm’s sensitivity to initial parameters can cause result variability, requiring careful tuning for consistent performance.	2021
^26^	Grey Wolf Optimizer (GWO)	2DOF-PI	GWO is known for its simplicity and effectiveness in various optimization tasks, making it a popular choice for AVR systems. It can adapt well to different optimization problems, providing flexibility in parameter tuning.	GWO may struggle with convergence speed in complex optimization scenarios, leading to longer computation times. Its performance can be inconsistent across different problem domains, requiring additional validation	2021
^27^	Modified artificial bee colony (ABC)	Low-order approximation (LOA) model of a fractional order PID (FOPID)	Enhanced dynamic performance, namely speed convergence, and is well suited for the AVR system while also performing better in terms of noise attenuation and disturbance rejection	Higher overshoot was observed compared to other optimization methods like the original ABC, PSO, and LUS.	2022

Techniques like GA and PSO were explored extensively for their potential to optimize controller fine-tuning. Nevertheless, each showcased certain limitations, such as GA’s tendency towards premature convergence and PSO susceptibility to getting trapped in local optima. To address these challenges, hybrid optimization techniques emerged, combining the strengths of multiple algorithms to enhance performance. For instance, the integration of deterministic and stochastic approaches within a hybrid framework has been shown to improve convergence rates while avoiding local minima, leading to more reliable solutions. As a result, these hybrid methods have proven to be more robust and effective in handling complex, high-dimensional optimization problems, particularly in scenarios where traditional methods might falter. A most recent example includes^28^, where the author not only proved the superiority of the hybrid method in comparison to the original single method in terms of efficiency in achieving better control outcomes under the same conditions but also exhibited superior performance in terms of steady and transient responses and improvements in overshoot maximum and steady-state error compared to existing methods including Biogeography-Based Optimization (BBO), Particle Swarm Optimization (PSO), Artificial Bee Colony (ABC), and Differential Evolution (DE). Another example is^29^, where the author used the fractional order proportional-integral-derivative (FOPID) controller with the hybrid particle swarm and grey wolf optimization (HPSGWO). The results of the simulation showed that the proposed HPSGWO algorithm surpassed both PSO and GWO algorithms in terms of the rise time, settling time, and overshoot.

The purpose of this paper is to present a novel hybrid technique between Harmony Search (HS) and Dwarf Mongoose Optimization (DMO) algorithms to enhance the AVR system controller performance. The proposed hybrid method guarantees robust performance through the balancing behavior of HS between exploration and exploitation rates and precise solutions through the high DMOA exploitation rate. The Harmony Search-Dwarf Mongoose Optimization algorithm (HS-DMOA) is presented against other well-known techniques, including Harmony Search (HS), Local Unimodal Sampling (LUS), and Teaching Learning Based Optimization (TLBO). Each optimization technique is applied to both the proportional-integral-derivative (PID) and proportional-integral-derivative-acceleration (PIDA) controllers, and the proposed hybrid HS-DMOA PIDA-based outperformed other techniques in both time and frequency domain indices. Moreover, the proposed HS-DMOA PIDA-based system proved its superiority under dynamic disturbance and different load disturbances.

The contribution of the paper can be summarized as follows:

1. Using a relatively new hybrid optimization technique to enhance the AVR system controllers performance.
2. A comparative study between the proposed technique and several optimization methods is carried out in time and frequency domains.
3. Robustness tests are carried out to validate the proposed technique under several types of disturbances.

The remaining parts of this paper are divided as follows: Sect. 2 contains the mathematical modeling, Sect. 3 is composed of the proposed optimization techniques, Sect. 4 consists of the results, Sect. 5 includes discussion and analysis of the results, and Sect. 6 consists of the validation through benchmark functions while, Sect. 6 discusses the conclusion and a summary of the results. Finally, Sect. 8 include the future work suggestions.

## Mathematical modeling

### Standalone AVR system

**Fig. 1:**
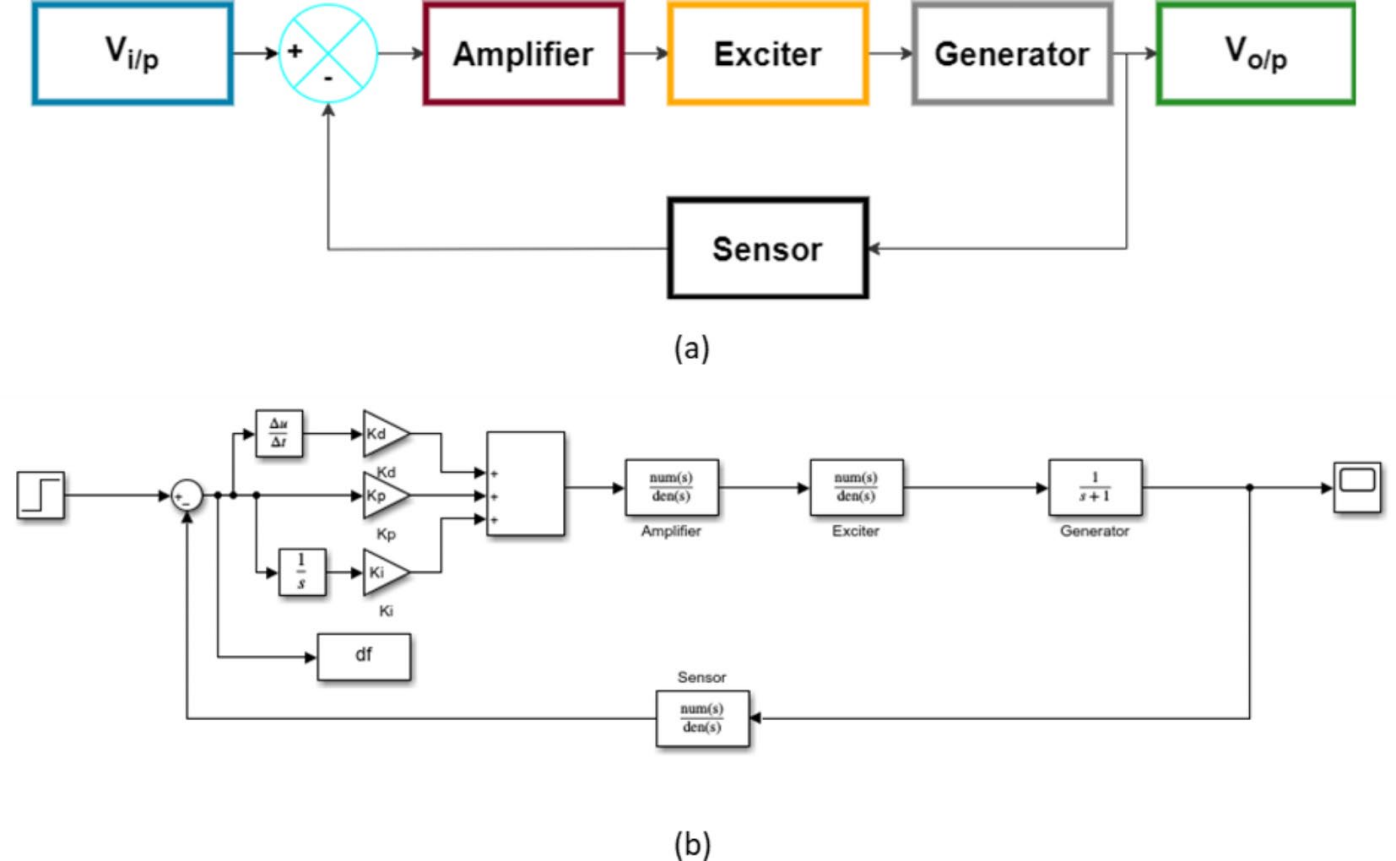
(**a**) Block diagram for AVR system without a controller. (**b**) Employed Simulink-based model for AVR system with a PID controller.

The mathematical representation of a standalone AVR system is shown in Eqs. (1–3)^30–34^—where each component is described in a block diagram structure in Fig. 1(a&b).

The Transfer Function (TF) is converted to the Laplace domain to be:

1 $$\:\mathrm{Y}\left(\mathrm{s}\right)=\frac{\mathrm{h}\left({{\upalpha\:}}_{4}\mathrm{s}+1\right)}{\mathrm{h}+\left({{\upalpha\:}}_{1}\mathrm{s}+1\right)\left({{\upalpha\:}}_{2}\mathrm{s}+1\right)\left({{\upalpha\:}}_{3}\mathrm{s}+1\right)\left({{\upalpha\:}}_{4}\mathrm{s}+1\right)}\mathrm{X}\left(\mathrm{s}\right)\:\:\:\:\:\:\:\:\:\:$$

Where $\:\mathrm{h}\:$, $\:{{\upalpha\:}}_{1}$, $\:{{\upalpha\:}}_{2}$, $\:{{\upalpha\:}}_{3}$, and $\:{{\upalpha\:}}_{4}$ compromises time constants of the amplifier, exciter, generator, and sensor, respectively. By applying inverse Laplace to the above equation, we get the following equation in the time domain:

2 $$\left[ {\mathop \prod \limits_{{{\mathrm{n}} = 1}}^{4} {{\upalpha }}_{{\mathrm{n}}} {\mathrm{D}}_{{\mathrm{t}}}^{4} + \mathop \sum \limits_{{{\mathrm{n}} = 1,{\mathrm{m}} > {\mathrm{n}},{\mathrm{k}} > {\mathrm{m}}}}^{4} {{\upalpha }}_{{\mathrm{n}}} {{\upalpha }}_{{\mathrm{m}}} {{\upalpha }}_{{\mathrm{k}}} {\mathrm{D}}_{{\mathrm{t}}}^{3} + \mathop \sum \limits_{{{\mathrm{n}} = 1,{\mathrm{m}} > {\mathrm{n}}}}^{4} {{\upalpha }}_{{\mathrm{n}}} {{\upalpha }}_{{\mathrm{m}}} {\mathrm{D}}_{{\mathrm{t}}}^{2} + \mathop \sum \limits_{{{\mathrm{n}} = 1}}^{4} {{\upalpha }}_{{\mathrm{n}}} {\mathrm{D}}_{{\mathrm{t}}} + \left( {1 + {\mathrm{h}}} \right)} \right]{\mathrm{y}}\left( {\mathrm{t}} \right) = {\mathrm{h}}\left( {{{\upalpha }}_{4} {\mathrm{s}} + 1} \right){\mathrm{~}} \times \left( {\mathrm{t}} \right)$$

Based on the prior equation, we can conclude the following fourth-order differential, characteristic equation to monitor and study the system’s stability features (including the bode plot, root locus, phase margin, and gain margin).

3 $$\prod \limits_{{{\mathrm{n}}=1}}^{4} {{{{\upalpha}}}_{\mathrm{n}}}{{\mathrm{s}}^4}+\mathop \sum \limits_{{{\mathrm{n}}=1,{\mathrm{m}}>{\mathrm{n}},{\mathrm{k}}>{\mathrm{m}}}}^{4} {{{{\upalpha}}}_{\mathrm{n}}}{{{{\upalpha}}}_{\mathrm{m}}}{{{{\upalpha}}}_{\mathrm{k}}}{{\mathrm{s}}^4}+\mathop \sum \limits_{{{\mathrm{n}}=1,{\mathrm{m}}>{\mathrm{n}}}}^{4} {{{{\upalpha}}}_{\mathrm{n}}}{{{{\upalpha}}}_{\mathrm{m}}}{{\mathrm{s}}^2}+\mathop \sum \limits_{{{\mathrm{n}}=1}}^{4} {{{{\upalpha}}}_{\mathrm{n}}}{\mathrm{s}}+\left( {1+{\mathrm{h}}} \right)= 0$$

To achieve a stable state, $\:\mathrm{h},\:{{\upalpha\:}}_{1},\:{{\upalpha\:}}_{2},\:{{\upalpha\:}}_{3},$ and $\:{{\upalpha\:}}_{4}$ must maintain the real parts of all the roots within the negative range.

### AVR system with Proportional-Integral-Derivative (PID) controller

The PID (Proportional-Integral-Derivative) controller is one of the earliest control methods known for its widespread due to its simple algorithm, strong robustness, and high reliability. A PID controller is a fundamental component of any control system due to its crucial role in ensuring optimal performance, efficiency, and stability in different applications across various industrial, research-based, and residential disciplines including programmable logic controllers (PLCs) and distributed control systems (DCSs)^35^. In essence, a PID controller’s focus is to maintain a desired setpoint by automatically adjusting terminal voltage based on the error between the desired setpoint and the actual measured value. The proportional term (Kp) addresses the current error, the integral term (Ki) reacts to past errors by integrating them over time, and the derivative term (Kd) anticipates future trends by evaluating the rate of change of the error^35^. The time constants and gains chosen for each block are listed below in Table 2. These three terms provide an extensive control strategy that enables the PID controller to reach stable and accurate control in a power system. Understanding the principles and functionalities of PID controllers is essential for engineers and researchers involved in control systems design and optimization, as it forms the basis for advanced control techniques and strategies aimed at enhancing system performance and responsiveness. The mathematical model of the controller in the Laplace domain is extensively discussed in Eqs. (4–8)^36–38^.

4 $$\:Y\left(s\right)\:\:={(\mathrm{k}}_{\mathrm{p}}+\frac{{\mathrm{k}}_{\mathrm{i}}}{\mathrm{s}}+{\mathrm{k}}_{\mathrm{d}}\mathrm{s})\:X\left(s\right)$$

where $\:\mathbf{X}\left(\mathbf{s}\right)$ and $\:\mathbf{Y}\left(\mathbf{s}\right)$ are the controller input and output respectively. The controller time-domain dynamic equation is defined by:

5 $$\:\mathrm{y}\left(\mathrm{t}\right)=\left({\mathrm{k}}_{\mathrm{p}}+{{\mathrm{K}}_{\mathrm{i}}\mathrm{D}}_{\mathrm{t}}^{-1}+{\mathrm{k}}_{\mathrm{d}}{\mathrm{D}}_{\mathrm{t}}\right)\mathrm{x}\left(\mathrm{t}\right)$$

**Fig. 2:**
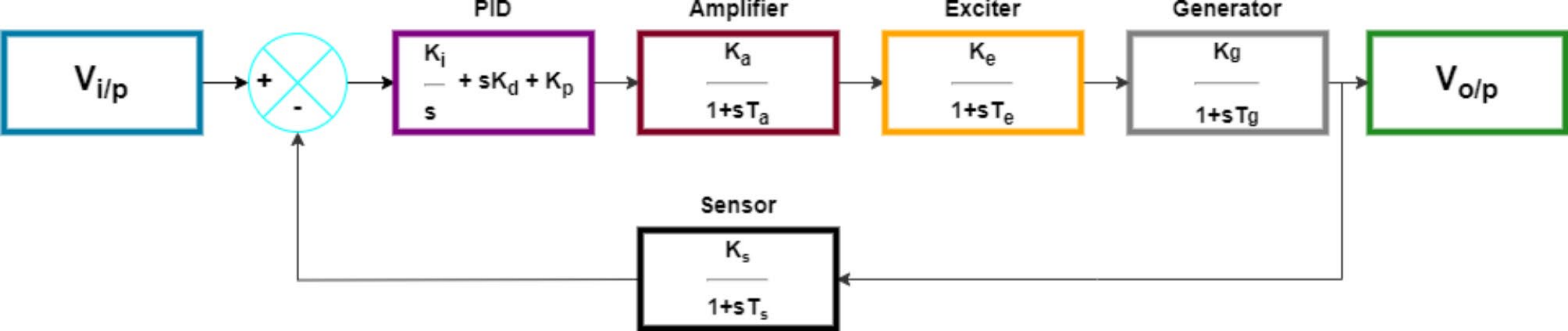
Block diagram of AVR system with PID controller model.

After connecting the PID controller, as shown in Fig. 2, the transfer function of the AVR system, as previously denoted in Eq. (1), can be described by:

6 $$\:Y\left(s\right)=\frac{h{(\mathrm{k}}_{\mathrm{d}}{\mathrm{s}}^{2}+{\mathrm{k}}_{\mathrm{p}}\mathrm{s}+{\mathrm{k}}_{\mathrm{i}})\left({\alpha\:}_{4}s+1\right)}{h{(\mathrm{k}}_{\mathrm{d}}{\mathrm{s}}^{2}+{\mathrm{k}}_{\mathrm{p}}\mathrm{s}+{\mathrm{k}}_{\mathrm{i}})+s\left({\alpha\:}_{1}s+1\right)\left({\alpha\:}_{2}s+1\right)\left({\alpha\:}_{3}s+1\right)\left({\alpha\:}_{4}s+1\right)}X\left(s\right)$$

and the AVR dynamic system governing differential equation in the time domain takes the form:

7 $$\begin{aligned} & \left[ {\mathop \prod \limits_{{{\mathrm{n}}=1}}^{4} {\alpha _n}{\mathrm{D}}_{{\mathrm{t}}}^{5}+\mathop \sum \limits_{{n=1,m>n,k>m}}^{4} {\alpha _n}{\alpha _m}{\alpha _k}{\mathrm{D}}_{{\mathrm{t}}}^{4}+\mathop \sum \limits_{{n=1,m>n}}^{4} {\alpha _n}{\alpha _n}{\mathrm{D}}_{{\mathrm{t}}}^{3}+(h{{\mathrm{k}}_{\mathrm{d}}}} \right. \\ & \quad \left. {+\sum\limits_{{{\mathrm{n}}=1}}^{4} {{\alpha _n}){\mathrm{D}}_{{\mathrm{t}}}^{2}+\left( {h{{\mathrm{k}}_{\mathrm{p}}}+1} \right){{\mathrm{D}}_{\mathrm{t}}}+h{{\mathrm{k}}_{\mathrm{i}}}} } \right]{\mathrm{y}}\left( {\mathrm{t}} \right) \\ & =h[{\alpha _4}{{\mathrm{k}}_{\mathrm{d}}}{\mathrm{D}}_{{\mathrm{t}}}^{3}+([{\alpha _4}{{\mathrm{k}}_{\mathrm{p}}}+{{\mathrm{k}}_{\mathrm{d}}}){\mathrm{D}}_{{\mathrm{t}}}^{2}+\left( {{\alpha _4}{{\mathrm{k}}_{\mathrm{i}}}+{{\mathrm{k}}_{\mathrm{p}}}} \right){{\mathrm{D}}_{\mathrm{t}}}+{{\mathrm{k}}_{\mathrm{i}}}]{\mathrm{~x}}\left( {\mathrm{t}} \right) \\ \end{aligned}$$

During system designing process, The PID-AVR system stability can be studied using the following system characteristic Eq. 

8 $$\mathop \prod \limits_{{{\mathrm{n}}=1}}^{4} {\alpha _n}{{\mathrm{s}}^5}+\mathop \sum \limits_{{n=1,m>n,k>m}}^{4} {\alpha _n}{\alpha _m}{\alpha _k}{{\mathrm{s}}^4}+\mathop \sum \limits_{{n=1,m>n}}^{4} {\alpha _n}{\alpha _n}{{\mathrm{s}}^3}+(h{{\mathrm{k}}_{\mathrm{d}}}+\mathop \sum \limits_{{{\mathrm{n}}=1}}^{4} {\alpha _n}){{\mathrm{s}}^2}+\left( {h{{\mathrm{k}}_{\mathrm{p}}}+1} \right){\mathrm{s}}+h{{\mathrm{k}}_{\mathrm{i}}}=0$$

**Table 2:** Conventional and used time constants and gains of elements.

Element name	Conventional range	Used value
Amplifier	10 $\:\le\:\:{K}_{a}\le\:$ 40 0.02 $\:\le\:\:{\tau\:}_{a}\le\:$ 0.1 (sec)	*K*_a_ = 10 *τ*_a_ = 0.1 (sec)
Field exciter	1 $\:\le\:\:{K}_{e}\le\:$ 10 0.4 $\:\le\:\:{\tau\:}_{e}\le\:$ 1 (sec)	*K*_e_ = 1 *τ*_e_ = 0.4 (sec)
AC generator	0.7 $\:\le\:\:{K}_{g}\le\:$ 1 1 $\:\le\:\:{\tau\:}_{g}\le\:$ 2 (sec)	*K*_g_ = 1 *τ*_g_ = 1 (sec)
Terminal voltage sensor	*K*_v_ = 1 0.01 $\:\le\:\:{\tau\:}_{v}\le\:$ 0.06 (sec)	*K*_v_ = 1 *τ*_v_ = 0.01 (sec)

### PIDA based AVR dynamic system model

The PIDA controller is one of most recent controllers studied by many researchers especially for the AVR system control. The PIDA’s mathematical representation is extensively discussed in: Eqs. (9–12)^7,39^.

The generalized transfer function of n-order type PIDA controller can be written as:

9 $$\:Y\left(s\right)=\frac{{\upbeta\:}{\mathrm{s}}^{\mathrm{i}}+{{\upbeta\:}}_{1}{\mathrm{s}}^{\mathrm{i}-1}+\dots\:+{{\upbeta\:}}_{\mathrm{i}}\:}{{\delta\:}_{0}{\mathrm{s}}^{\mathrm{i}}+{{\updelta\:}}_{1}{\mathrm{s}}^{\mathrm{i}-1}+\dots\:+{{\updelta\:}}_{\mathrm{i}}}X\left(s\right)\:$$

where $\:\left({\boldsymbol{\upbeta\:}}_{0},\:{\boldsymbol{\upbeta\:}}_{1},\dots\:,{\boldsymbol{\upbeta\:}}_{\mathbf{i}},{\boldsymbol{\updelta\:}}_{0},\:{\boldsymbol{\updelta\:}}_{1},\dots\:,{\boldsymbol{\updelta\:}}_{\mathbf{i}}\right)$ are the controller designing parameters.

**Fig. 3:**
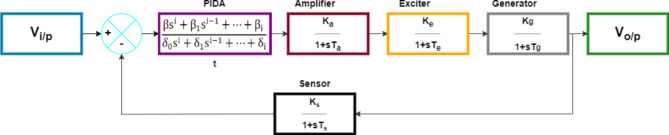
Block diagram of AVR system with PIDA controller model.

Selecting the parameters of the controller for an optimal control system is the main concern of many studies where different types of metaheuristic optimization techniques are employed. The AVR system modelling equation under this controller, which is connected as in Fig. 3. To investigate how the system complexity increased when this controller was used, we will first obtain the system governing differential equation of the second order PIDA type with *n* = 2, as in Eq. (10).

10 $$Y\left( s \right) = \frac{{h\left( { {\upbeta }_{0} {\mathrm{s}}^{{\mathrm{i}}} + {\upbeta }_{1} {\mathrm{s}}^{{{\mathrm{i}} - 1}} + \cdots + {\upbeta }_{{\mathrm{i}}} } \right)\left( { {\upalpha }_{4} {\mathrm{s}} + 1} \right)}}{{\left( { {\updelta }_{0} {\mathrm{s}}^{{\mathrm{i}}} + {\updelta }_{1} {\mathrm{s}}^{{{\mathrm{i}} - 1}} + \cdots + {\updelta }_{{\mathrm{i}}} } \right)~\left( { {\upalpha }_{1} {\mathrm{s}} + 1} \right)\left( { {\upalpha }_{2} {\mathrm{s}} + 1} \right)\left( { {\upalpha }_{3} {\mathrm{s}} + 1} \right)\left( { {\upalpha }_{4} {\mathrm{s}} + 1} \right) + h\left( { {\upbeta }_{0} {\mathrm{s}}^{{\mathrm{i}}} + {\upbeta }_{1} {\mathrm{s}}^{{{\mathrm{i}} - 1}} + \cdots + {\upbeta }_{{\mathrm{i}}} } \right)}}X\left( s \right)$$

After taking the inverse Laplace transform to Eq. (10), and making all possible simplifications, the dynamic system differential equation is written as

11 $$\begin{aligned} & \left[ {\prod\limits_{{n = 1}}^{4} {{\updelta }_{0} \alpha _{n} D_{t}^{6} + } } \right.\left( {\mathop \sum \limits_{{n = 1,m > n,k > m}}^{4} {\updelta }_{0} \alpha _{n} \alpha _{m} \alpha _{k} + \mathop \prod \limits_{{n = 1}}^{4} {\updelta }_{1} \alpha _{n} } \right)D_{t}^{5} + \left( {\mathop \sum \limits_{{n = 1,m > n}}^{4} {\updelta }_{0} \alpha _{n} \alpha _{n} + \mathop \sum \limits_{{n = 1,m > n,k > m}}^{4} {\updelta }_{1} \alpha _{n} \alpha _{m} \alpha _{k} + \mathop \prod \limits_{{n = 1}}^{4} {\updelta }_{2} \alpha _{n} } \right)D_{t}^{4} \\ & \quad + \left( {\mathop \sum \limits_{{n = 1}}^{4} {\updelta }_{0} \alpha _{n} + \mathop \sum \limits_{{n = 1,m > n}}^{4} {\updelta }_{1} \alpha _{n} \alpha _{m} + \mathop \sum \limits_{{n = 1,m > n,k > m}}^{4} {\updelta }_{2} \alpha _{n} \alpha _{m} \alpha _{k} } \right)D_{t}^{3} \\ & \quad \left. { + \left( {{\updelta }_{0} + \mathop \sum \limits_{{n = 1}}^{4} {\updelta }_{1} \alpha _{n} + \mathop \sum \limits_{{n = 1,m > n}}^{4} {\updelta }_{2} \alpha _{n} \alpha _{n} + h{\upbeta }_{0} } \right)D_{t}^{2} + \left( {{\updelta }_{1} + \mathop \sum \limits_{{n = 1}}^{4} {\updelta }_{2} \alpha _{n} + h{\upbeta }_{1} } \right)D_{t} + {\updelta }_{2} + h{\upbeta }_{2} } \right]y\left( t \right) \\ & = h[\alpha _{4} {\upbeta }_{0} D_{t}^{3} + \left( {[\alpha _{4} {\upbeta }_{1} + {\upbeta }_{0} )D_{t}^{2} + \left( {\alpha _{4} {\upbeta }_{2} + {\upbeta }_{1} } \right)D_{t} + {\upbeta }_{2} } \right]~x\left( t \right) \\ \end{aligned}$$

which is a sixth-order linear ordinary differential equation. The stability of the PIDA based AVR system can be studied using the system characteristic equation defined by:

12 $$\begin{aligned} & \left[ {\prod\limits_{{n = 1}}^{4} {\updelta _{0} \alpha _{n} s^{6} + \left( {\mathop \sum \limits_{{n = 1,m > n,k > m}}^{4} \updelta _{0} \alpha _{n} \alpha _{m} \alpha _{k} + \mathop \prod \limits_{{n = 1}}^{4} \updelta _{1} \alpha _{n} } \right)s^{5} + \left( {\mathop \sum \limits_{{n = 1,m > n}}^{4} \updelta _{0} \alpha _{n} \alpha _{n} + \mathop \sum \limits_{{n = 1,m > n,k > m}}^{4} \updelta _{1} \alpha _{n} \alpha _{m} \alpha _{k} + \mathop \prod \limits_{{n = 1}}^{4} \updelta _{2} \alpha _{n} } \right)s^{4} } } \right. \\ & \quad + \left( {\mathop \sum \limits_{{n = 1}}^{4} \updelta _{0} \alpha _{n} + \mathop \sum \limits_{{n = 1,m > n}}^{4} \updelta _{1} \alpha _{n} \alpha _{m} + \mathop \sum \limits_{{n = 1,m > n,k > m}}^{4} \updelta _{2} \alpha _{n} \alpha _{m} \alpha _{k} } \right)s^{3} \\ & \left. {\quad + \left( {\updelta _{0} + \mathop \sum \limits_{{n = 1}}^{4} \updelta _{1} \alpha _{n} + \mathop \sum \limits_{{n = 1,m > n}}^{4} \updelta _{2} \alpha _{n} \alpha _{n} + h\upbeta _{0} } \right)s^{2} + \left( {\updelta _{1} + \mathop \sum \limits_{{n = 1}}^{4} \updelta _{2} \alpha _{n} + h\upbeta _{1} } \right)s + \updelta _{2} + h\upbeta _{2} } \right] = 0 \\ \end{aligned}$$

### Proposed optimization techniques

### Harmony search algorithm

Harmony Search (HS) algorithm is another metaheuristic technique that belongs to the category of swarm-based intelligence optimization algorithms. HS is inspired by the improvisation process of musicians in a jazz band, where they seek harmonious sound by trying various combinations of musical notes. In HS, each solution vector is akin to a musician’s improvisation. The HS algorithm is based on the natural musical performance processes of inspecting for the best state of music harmony. By mimicking musical improvisation, HS effectively balances exploration and exploitation in the search space, leading to robust performance in solving various optimization problems across engineering and scientific applications. Intensification and diversification of the set of solutions are the main causes behind the robust efficiency and widespread adoption of HS as a metaheuristic algorithm^40^. The method was readily discussed in^41–43^.

The steps of HS algorithm are given below:

*STEP I: Initialization of the optimization problem*.

Algorithm parameters are established as shown in Eq. (13)^44^:

13 $$\begin{aligned} & Min.{\mathrm{~}}f\left( {{h_i}} \right){\mathrm{~}} \\ & subj.{\mathrm{~}}to{\mathrm{~~}}{h_i} \in {\mathrm{~}}{H_i};i=1,2, \ldots ,N. \\ \end{aligned}$$

where *f* ($\:{h}_{i}$) is the objective function to be optimized, and *N* are the total number of $\:{h}_{i}$ continuous decision variable$\:.{\boldsymbol{H}}_{i}$ is the range of possible values for each $\:{h}_{i}$^41^. In addition, the control parameters of HS are also specified in this step, which are:

**Harmony Memory** (HM): The decision variables and their corresponding objective function values are stored in a matrix called Harmony memory.

**Harmony Memory Size** (HMS): Total number of solution vectors presented in HM.

**Harmony Memory Consideration Rate** (HMCR): HMCR ∈ [0, 1] and is the probability of selecting a harmony present in the HM.

**Pitch Adjusting Rate** (PAR): PAR ∈ [0, 1] and is the degree of adjustment.

**Randomization** (rand): utilized to increase the diversity of the solution, the MATLAB-based function is defined as is a uniformly distributed random number rand ∈ [0, 1].

probability of randomization: 1−.

**Number of Improvisations** (NI): The number of solutions generated per iteration.

**Bandwidth** (BW): Usually assigned as an arbitrary distance, the bandwidth is the number of changes allowed in a single pitch-adjustment round. Typically, bandwidth values are kept relatively low for better calibrating and exploration.

***h***_***LB***_, ***h***_***UB***_: Lower and higher bounds of decision variables.

*STEP II: Harmony Memory matrix formation*.

The random vectors are developed (*h*^1^, *h*^2^, *h*^3^, …, *h*^*HMS*^), as many as Harmony Memory Size (*HMS*), and store them in the Harmony Memory (*HM*) matrix as in Eq. (14):^45^.

14 $$\left\{ {\begin{array}{*{20}l} {h_{1}^{1} } & \cdots & {h_{n}^{1} } \\ \vdots & \ddots & { \vdots 45} \\ {h_{1}^{{hms}} } & \cdots & {h_{n}^{{hms}} } \\ \end{array} \left| {\begin{array}{*{20}l} {f\left( {h^{1} } \right)} \\ \vdots \\ {f\left( {h^{{hms}} } \right)} \\ \end{array} } \right.} \right\}$$

*STEP III: Improvising a new harmony*.

In this phase, a new harmony vector [$\:\overrightarrow{{\boldsymbol{H}}^{new}}=({h}_{1}^{new},{h}_{2}^{new},\dots\:,{h}_{n}^{new})\:$] is generated based on three rules:

### Random selection

The $\:{i}^{th}$ decision variable value of the new solution $\:{h}^{new}$ could be chosen randomly where $\:{h}_{LB}^{new}\:\:\le\:\:{h}_{i}^{new}\:\le\:\:{h}_{UB}^{new}$ with probability of (*1 – HMCR*) %. In addition, random selection is also applied for initiating the harmony memory. as in Eq. (15):^46^.

$$\:if\:\left(rand\:\in\:\:\left(\mathrm{0,1}\right)\ge\:\:HMCR\:\right)then$$

15 $$\:{h}_{i}^{new}\:=\:rand(\:{h}_{i}^{LB},\:{h}_{i}^{UB}\:)$$

### Memory consideration

The HS technique could choose a new global optimum, or solution, $\:{h}^{new}$ by randomly selecting it from the harmony memory with probability (*HMCR*) % using as in Eq. (16):^47^.

16 $$\:\left\{\begin{array}{l}if\:\left(rand\: \epsilon \:\left(\mathrm{0,1}\right)\le\:HMCR\right)\:then\\\:{h}^{new}\:\in\:HM\end{array}\right.$$

For instance, a probability of HMCR = 0.60 indicates that the HS algorithm will choose the decision variable value from historically stored values in the HM with a 60% probability or from the entire possible range with a 40% probability.

### Pitch adjustment

Every $\:{h}^{new}$ component obtained by the memory consideration is further examined to determine whether it should be pitch adjusted or not according to its neighboring values using the parameter (PAR) as in Eq. (17):^48^.

17 $$\begin{aligned} & if~\left( {rand~ \in ~\left( {0,1} \right) \leqslant ~PAR} \right)then \\ & ~h_{i}^{{new}}~=~~h_{i}^{{new}}~+~BW*~rand\left( { - 1,1} \right)~ \\ \end{aligned}$$

*STEP IV*: Harmony Memory Update.

This is considered the selection step of the technique: If the new harmony vector $\:\overrightarrow{{\boldsymbol{H}}^{new}}=\:({h}_{1}^{new},{h}_{2}^{new},\dots\:,{h}_{N}^{new}\:)$ is more favorable than the current most favorable harmony in the HM, based on the objective function’s relative values, the $\:\overrightarrow{{\boldsymbol{H}}^{new}}$ is included in the HM instead of the old harmony as in Eq. (18):^49^.

18 $$\begin{aligned} & if~f\left( {{h^{new}}} \right)<~f\left( {{h^{old}}~} \right)then \\ & HM\left( {old,:} \right)=~{h^{new}} \\ \end{aligned}$$

**Fig. 4:**
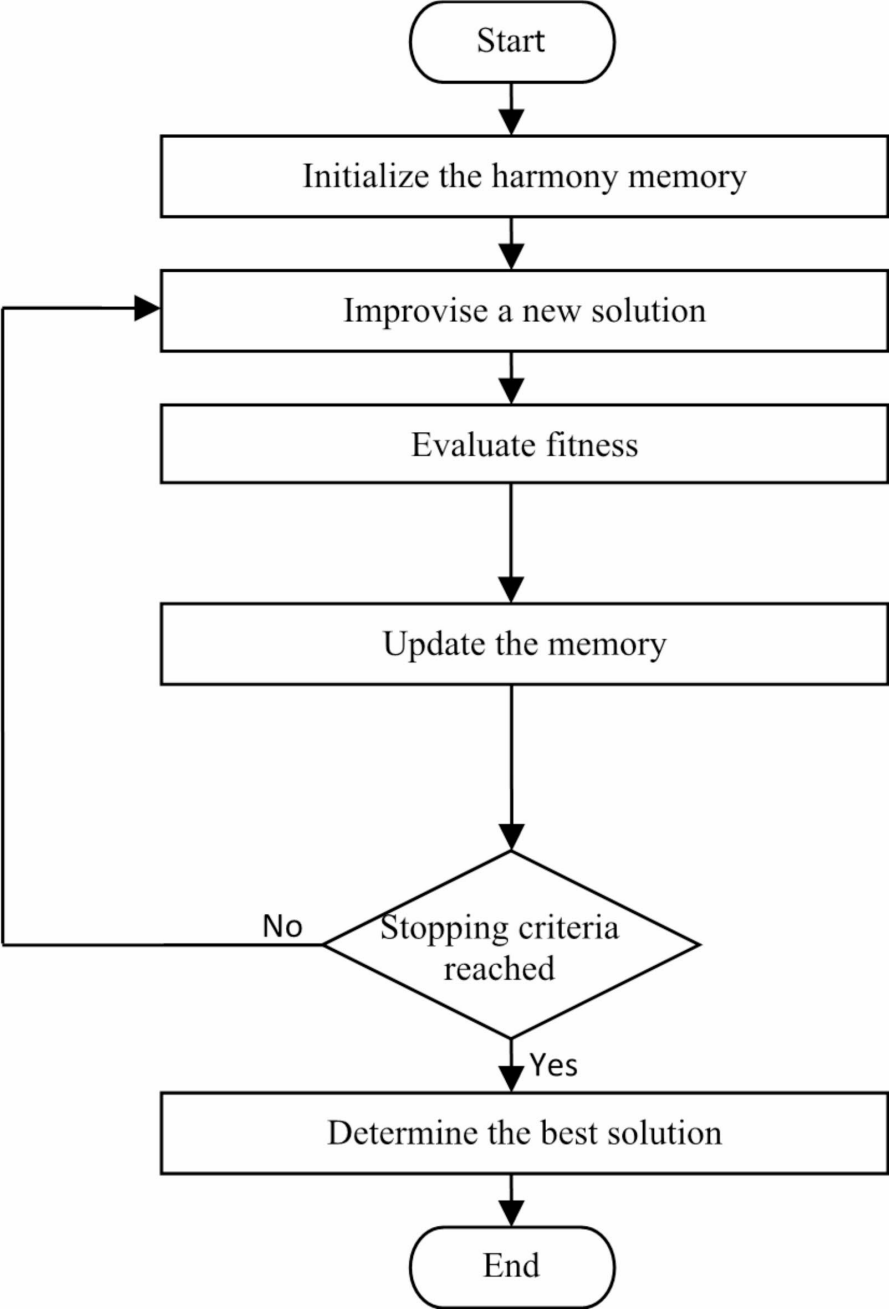
Flowchart of harmony search.

*STEP V*: STEP IV is recycled until we hit the maximum number of iterations. The pseudo code of HS is shown in Algorithm 1, while the HS flowchart is shown in Fig. 4.

**Algorithm 1:**
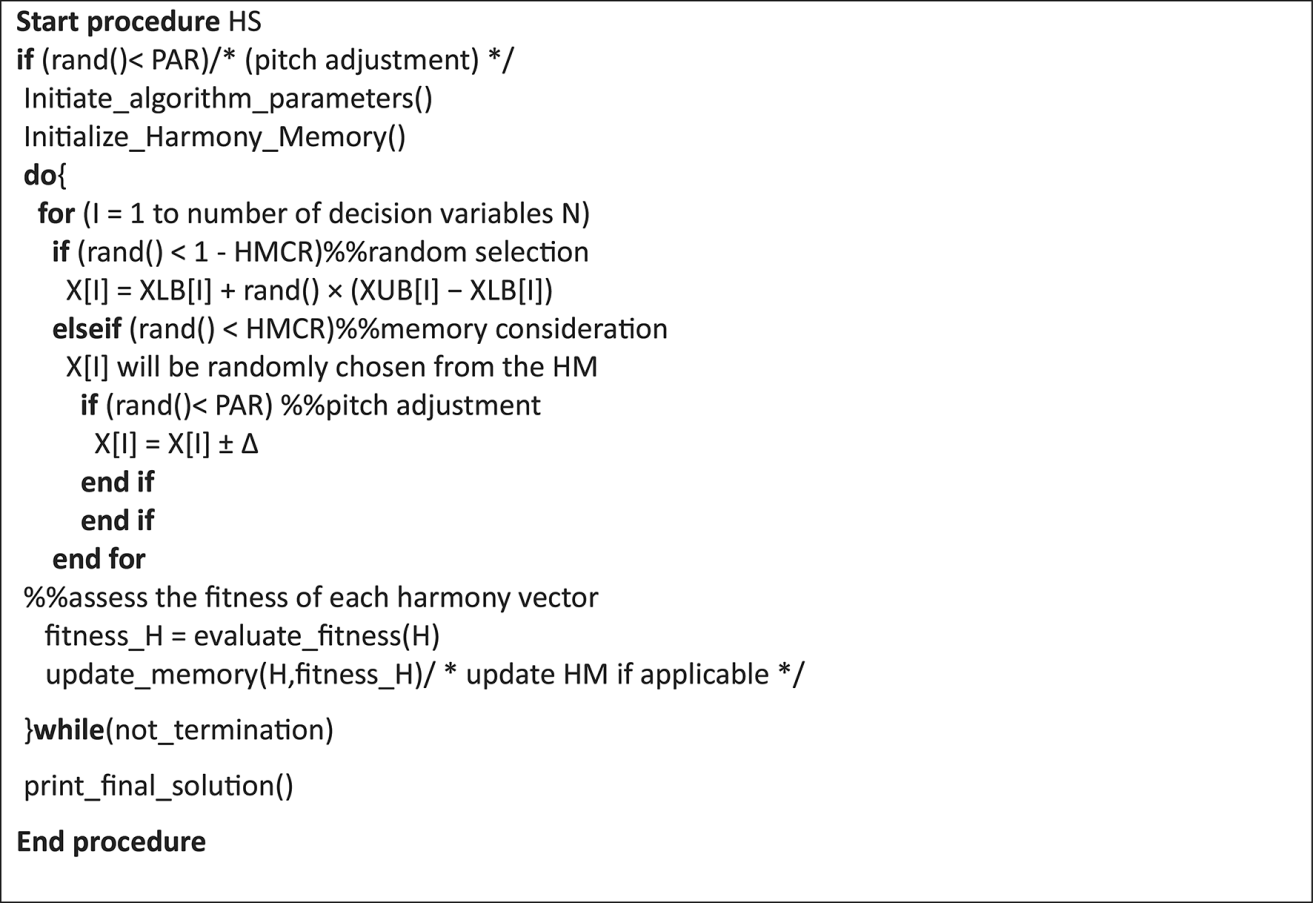
Pseudo-code of the HS.

### Dwarf Mongoose optimization algorithm

In essence, the DMOA is a bio-inspired optimization technique that draws inspiration from the behavior and social interactions of dwarf mongooses and small mongooses in Africa^50^. These small mammals are known for their highly organized social structure and cooperative behaviors, which are crucial for their survival in the wild. One key aspect of their behavior is cooperative foraging, where dwarf mongooses forage in groups, communicating and sharing information about food sources. This collective searching strategy ensures that the group efficiently explores their environment to find and exploit food resources. In addition to foraging, some members of the mongoose group act as sentinels, keeping watch for predators and alerting the group to danger. This role rotates among members to ensure that everyone contributes to the group’s safety, enhancing the algorithm’s flexibility and robustness. Moreover, the division of labor among dwarf mongooses, such as foraging, sentinel duty, and grooming, is dynamic and flexible, allowing the group to respond effectively to changing environmental conditions. By incorporating these social and cooperative behaviors, the DMOA is designed to explore and exploit the search space more effectively, balancing exploration with exploitation^51^. The method was readily discussed in:^10^.

Generally, swarm intelligence-based optimization techniques commence with random initialization of the herd or tribe. Then, according to the intensification and diversification rules, each solution, or individual, start assembling around the global best optima, or the alpha female. Similarly, the DMOA optimization begins with initializing the candidate population of the mongooses (M), as in Eq. (19). Population is constructed stochastically in the given problem’s UL (Upper Limit) and LL (Lower Limit)^10^.

19 $$M = \left[ {\begin{array}{*{20}l} {m_{{1,1}} } \hfill & {m_{{1,2}} } \hfill & \cdots \hfill & {m_{{1,y - 1}} } \hfill & {m_{{1,y}} } \hfill \\ {~m_{{2,1}} } \hfill & {m_{{2,2}} } \hfill & \cdots \hfill & {m_{{2,y - 1}} } \hfill & {m_{{2,y}} } \hfill \\ \vdots \hfill & \vdots \hfill & {m_{{p,z}} } \hfill & \vdots \hfill & \vdots \hfill \\ {m_{{x,1}} } \hfill & {m_{{x,2}} } \hfill & \cdots \hfill & {m_{{x,y - 1}} } \hfill & {m_{{x.y}} } \hfill \\ \end{array} } \right] $$$$

Where *M* represents the assembly of the mongoose candidates’ present population that are generated randomly using Eq. (19), indicates the position of the $\:{z\:}^{th}\:$dimension of the $\:{p}^{th}$ population, $\:x$ indicates the total population size, and $\:y$ is the dimension of the problem.

The MATLAB built-in function *unifrnd* in Eq. (20), is used as a tool to generate a stream of uniformly distributed random numbers. Furthermore, *D* reflets the number of decision variables or the mongooses’ features, while *LB* and *UB* are the problem’s lower and upper limits, respectively^52^.

20 $$\:{m}_{p,z}\:=unifrnd(LB,\:UB,\:D)$$

The optimum result in every preparation is considered the best-fit results so far. Subsequently, after the initialization finishes, the degree of fitness, described in Eq. (21), for every member of the mongoose herd is determined. The member with the highest fit probability is chose as the alpha female^53^.

21 $$\:\alpha\:\:=\:\frac{{Fit}_{n}}{{\sum\:}_{n=1}^{x}{Fit}_{n}}$$

The actual tally of mongooses in *α* coincided to $\:x-bb$, where the number of babysitters *bb*. The vocalization of the dominant female *P*, which stands for the *peep* sound that keeps the herd within the intended path. The solution’s updated method was computed using Eq. (22):^54^.

22 $$\:{S}_{n+1}\:={\:S}_{n}\:+\:\varnothing\:*P$$

Here, ∅ stands for the distributed random number. The sleeping mound $\:\partial\:$ was computed for every reiteration Eq. (23):^50^.

23 $$\:{\partial\:}_{n}\:=\frac{{\:Fit}_{n+1}\:-{Fit}_{n}}{max\left\{\left|{Fit}_{n+1},\:{Fit}_{n}\right|\right\}}$$

The average of $\:{\partial\:}_{n}$ was calculated Eq. (24):^55^.

24 $$\:\gamma\:\:=\:\frac{\sum\:_{n=1}^{x}{\partial\:}_{n}}{x}$$

The algorithm moved to the following group, or phase, when the babysitter criteria was met.

### Scout group

During this phase, if the family forages far enough, then a new sleeping mound will be discovered—this was calculated using Eq. (25)^56^:

25 $$\:\:{S}_{n+1}=\left\{\begin{array}{l}{S}_{n}-CVM*\:\varnothing\:*rand\:\left[{S}_{n}-\overrightarrow{{M}_{v}}\right]\:\:\:\:\:\:\:\:\:\:if\:{\theta\:}_{n+1}<{\theta\:}_{n}\\\:{S}_{n}+CVM*\:\varnothing\:*rand\:\left[{S}_{n}-\overrightarrow{{M}_{v}}\right]\:\:\:\:\:\:\:\:\:\:\:\:\:\:\:\:\:else\end{array}\right.\:\:$$

Here, the rand value formed between [0, 1]. CVM was a parameter for controlling the collective volitive movement of the mongoose group and $\:{\overrightarrow{M}}_{v}$ was the movement vector.

They were both calculated using Eq. (26) and Eq. (27):^57,58^.

26 $$\:CVM\:=\:{\left(1-\:\frac{i}{{M\mathrm{a}\mathrm{x}}_{i}}\right)}^{\left(\frac{2i}{{\mathrm{M}\mathrm{a}\mathrm{x}}_{i}}\right)}$$

27 $$\:\:{\overrightarrow{M}}_{v}=\:{\sum\:}_{n=1}^{x}\frac{{S}_{n}\times\:\:{\partial\:}_{n}\:}{{S}_{n}}$$

**Fig. 5:**
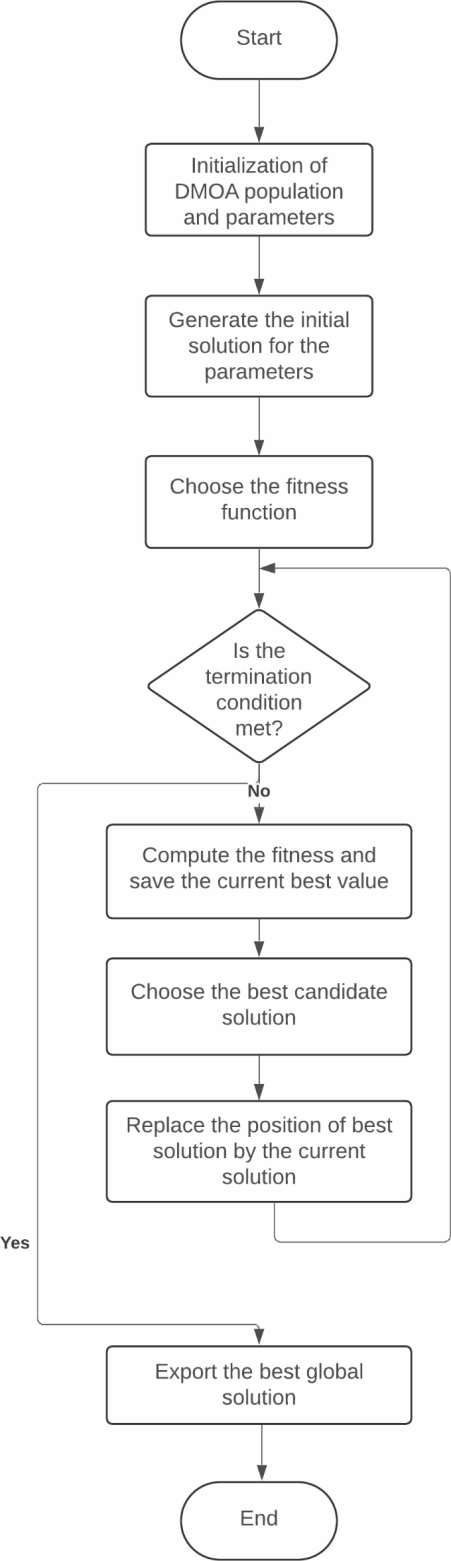
Flowchart of Dwarf Mongoose.

The babysitters are the subordinate party that remains with the juveniles. To help the alpha female, babysitters are replaced on a typical basis, while the rest of the tribe preforms daily hunting missions and other foraging tasks. The pseudocode of the DMOA is presented in Algorithm 2. The flowchart for the DMOA is shown below in Fig. 5^59^.

**Algorithm 2:**
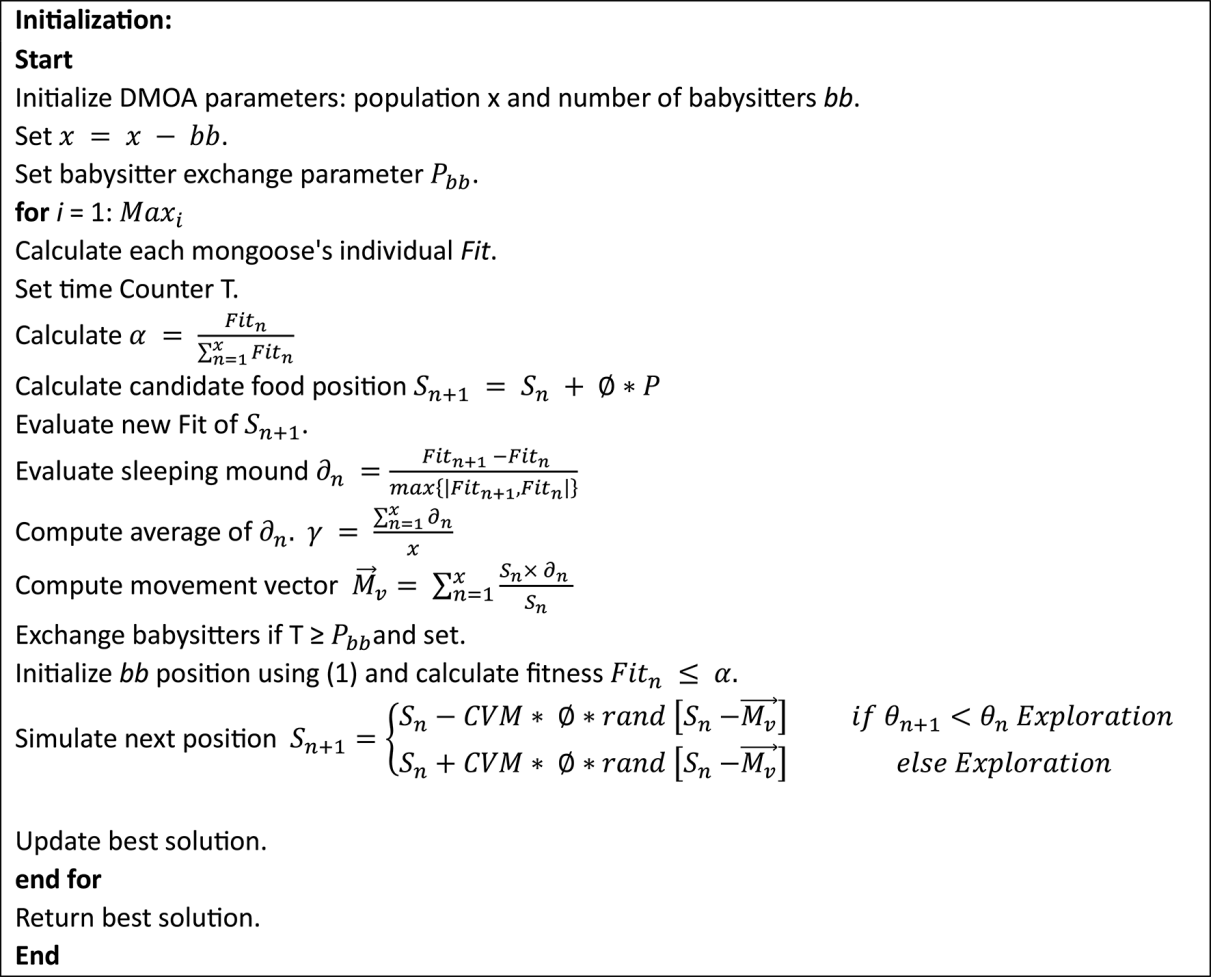
Pseudo-code of the DMOA.

The proposed hybrid HS-DMOA is carried out by combining between the high exploration rate of the Harmony Search (HS) algorithm and the high exploitation rate of the Dwarf Mongoose optimization algorithm (DMOA) through using the output of the HS optimization as the initials for the DMOA optimization. It is worth noting that the main limitation of the proposed hybrid algorithm is the extended time of operation; However, because the time of operation affects only online optimization, it doesn’t affect our case as it is an offline optimization.

## Results

In the first case, a step disturbance is applied to the proposed HS-DMOA, then a comparison with different optimization techniques is carried out on time and frequency domains. After that robustness test is carried out to prove the reliability of the proposed technique. Moreover, the proposed HS-DMOA PIDA based are examined against different disturbances to prove its superiority.

### PID time response analysis

**Fig. 6:**
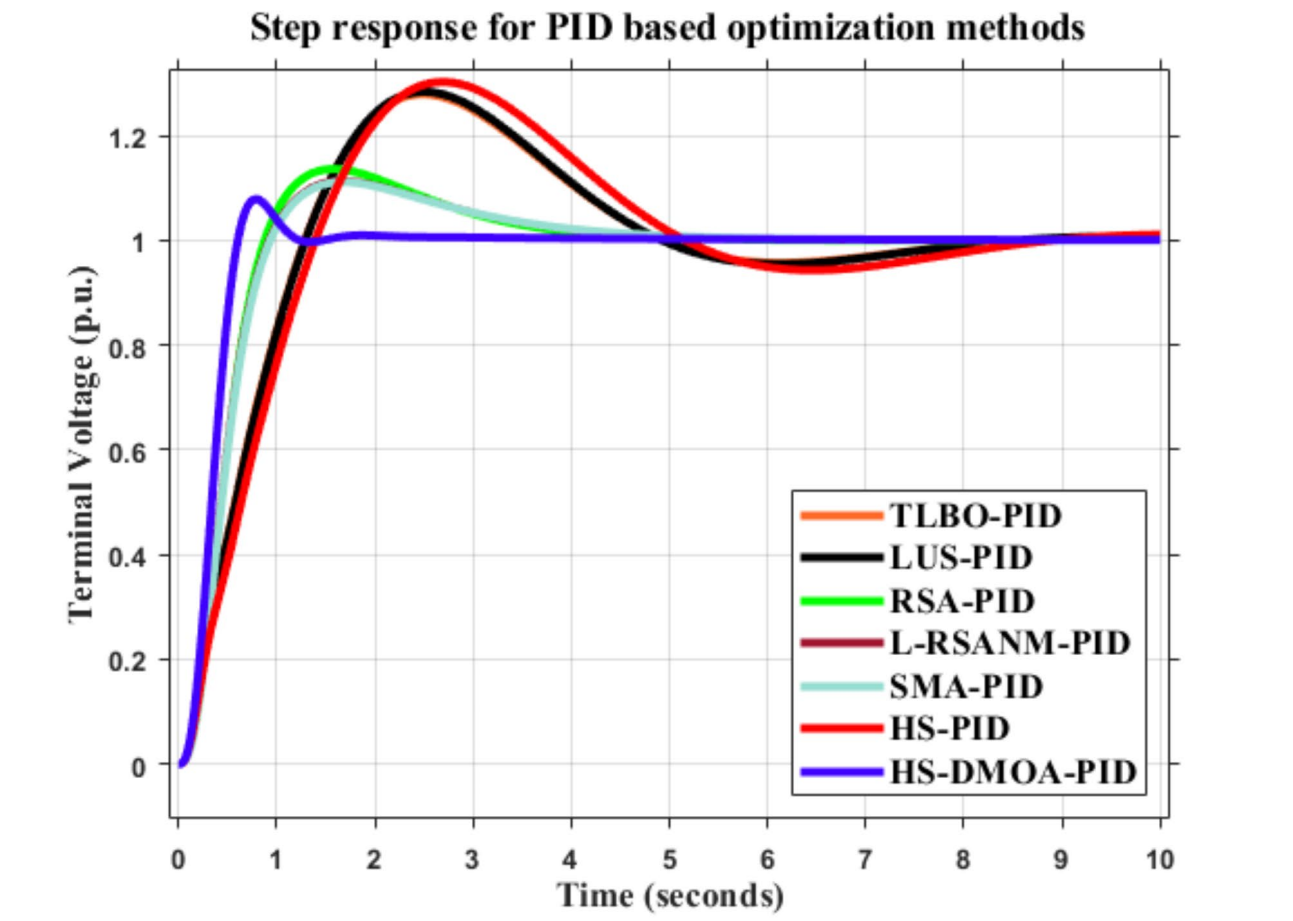
Output voltage of AVR system using HS-DMOA-PID controller vs. other optimization techniques.

The AVR is applied with a step response of one Per Unit (p.u.). The performance of the output voltage in the system for the PID HS-DMOA is demonstrated below in Fig. 6.

**Fig. 7:**
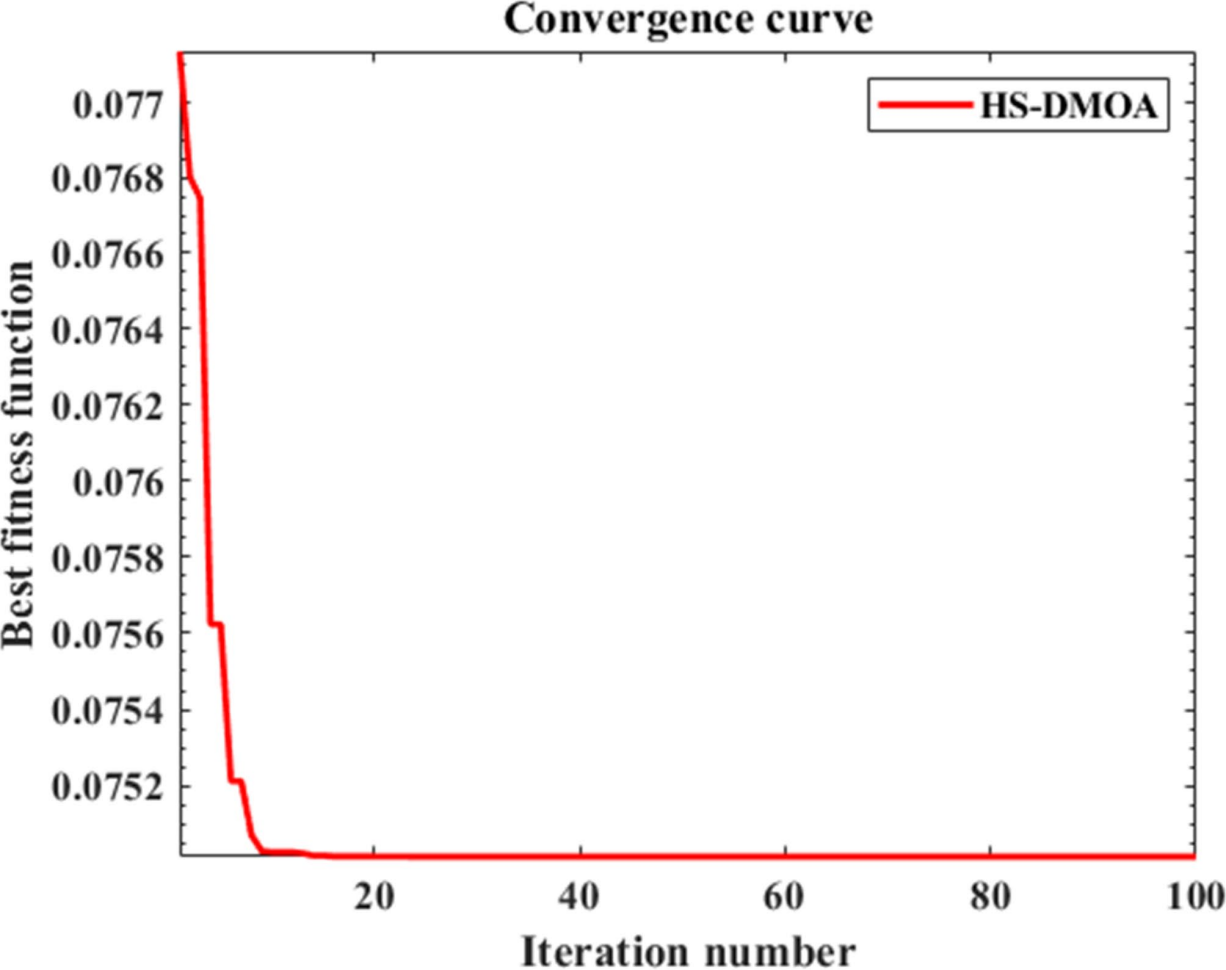
Convergence curve of HS-DMOA.

The method proved, mainly due to its rigid damping frequency, its superiority in possessing a more decisive nature, fewer oscillation cycles, and a more robust and faster response when it comes to reaching the steady-state error. In addition, the PID HS-DMOA technique proved lower sensitivity to disturbances, shorter settling time, and better overall system stability due to a decrease in the maximum overshoot. Figure 7 shows the progress of the HS-DMOA algorithm towards finding the optimal solution over one hundred iterations.

Based on Fig. 7 it is concluded that the HS-DMOA reaches early convergence to optimum and does decline rapidly.

The response of several other optimization techniques is also observed in Table 3. The table below illustrates the adaptability of the system incorporating the PID HS-DMOA optimization technique,

**Table 3:** Optimization techniques response using PID controller.

Optimization method	*K*_*p*_	*K*_*i*_	*K*_*d*_	Maximum overshoot	Rise time (sec.)	Settling time (sec.)
HS-DMOA-PID	1.0040	0.3347	0.2009	1.08	0.3	1.1
HSA-PID^6^	0.8683	0.9325	0.9419	1.3	1.03	8.1
LUS-PID^6^	0.9519	0.9997	0.8993	1.28	0.95	7.6
TLBO-PID^6^	0.9685	1	0.89825	1.28	0.94	7.5
SMA-PID^61^	0.6173	0.4166	0.2035	1.11	0.57	4.1
RSA-PID^62^	0.6105	0.4692	0.1988	1.14	0.55	3.8
L-RSANM-PID^62^	0.6294	0.4275	0.2079	1.11	0.56	4.1

PID HS-DMOA scored higher in terms of stability and reaction to disturbance indicators - lower maximum overshoot, lower settling time, and rise time, than other PID based techniques, including the HS, LUS, and TLBO. Numerically speaking, the PID HS-DMOA has: 18.5%, 18.5%, 20.37%, 2.77%, 5.55%, and 2.77% less overshoot, 581.81%, 590.9%, 636.36%, 272.72%, 245.45%, and 272.72% less settling time, and 213.33%, 216.66%, 243.33%, 90%, 83.33%, and 86.66% less rise time than TLBO, LUS, HS, slime mould algorithm (SMA), Reptile Search Algorithm (RSA), and Lévy flight-based reptile search and Nelder–Mead (L-RSANM), respectively.

### PID root locus

**Fig. 8:**
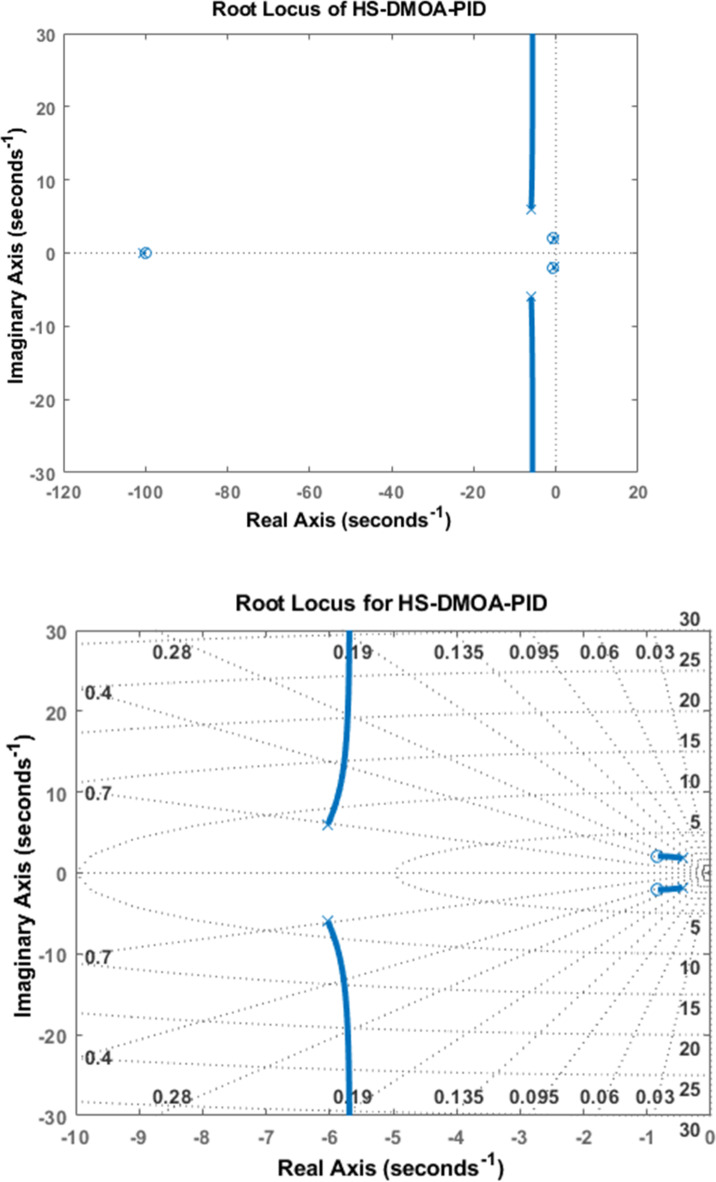
Root locus diagram of HS-DMOA-PID controller.

The root locus diagrams of HS-DMOA on the AVR system with a PID controller are shown in Fig. 8 below. They are obtained for the transfer functions Eq. (6).

The root locus indicates that the HS-DMOA PID controller is stable, because of the presence of all the roots are on the negative side of the Real Axis in the S-plane.

Table 4 demonstrates how the eigenvalues and damping ratio are retrieved from the root locus diagram of each optimization method. This table also shows the poles and damping ratios for the other techniques, such as TLBO, LUS, and HSA, incorporating the PID controller.

**Table 4:** Poles and damping ratio of optimization techniques using PID controller.

HS-DMOA-PID	Pole	-100	-3.96 + 4.80i	-2.91-5.28i	-2.52 + 0.32i	-0.295
	Damping ratio	1	0.6361	0.6361	0.9918	0.9918
LUS^6^	Pole	-102	-5.03-14.7i	-5.03-14.7i	-0.5 + 0.88i	-0.5 + 0.88i
	Damping ratio	1	0.323	0.323	0.502	0.502
HSA^6^	Pole	-103	-5.04 + 15.1i	-5.04 + 15.1i	-0.45 + 0.85i	-0.45 + 0.85i
	Damping ratio	1	0.316	0.316	0.465	0.465
TLBO-PID^6^	Pole	-102	-5.02 + 14.7i	-5.02-14.7i	-0.52 + 0.86i	-0.52-0.86i
	Damping ratio	1	0.323	0.323	0.511	0.511

### PID bode diagram

**Fig. 9:**
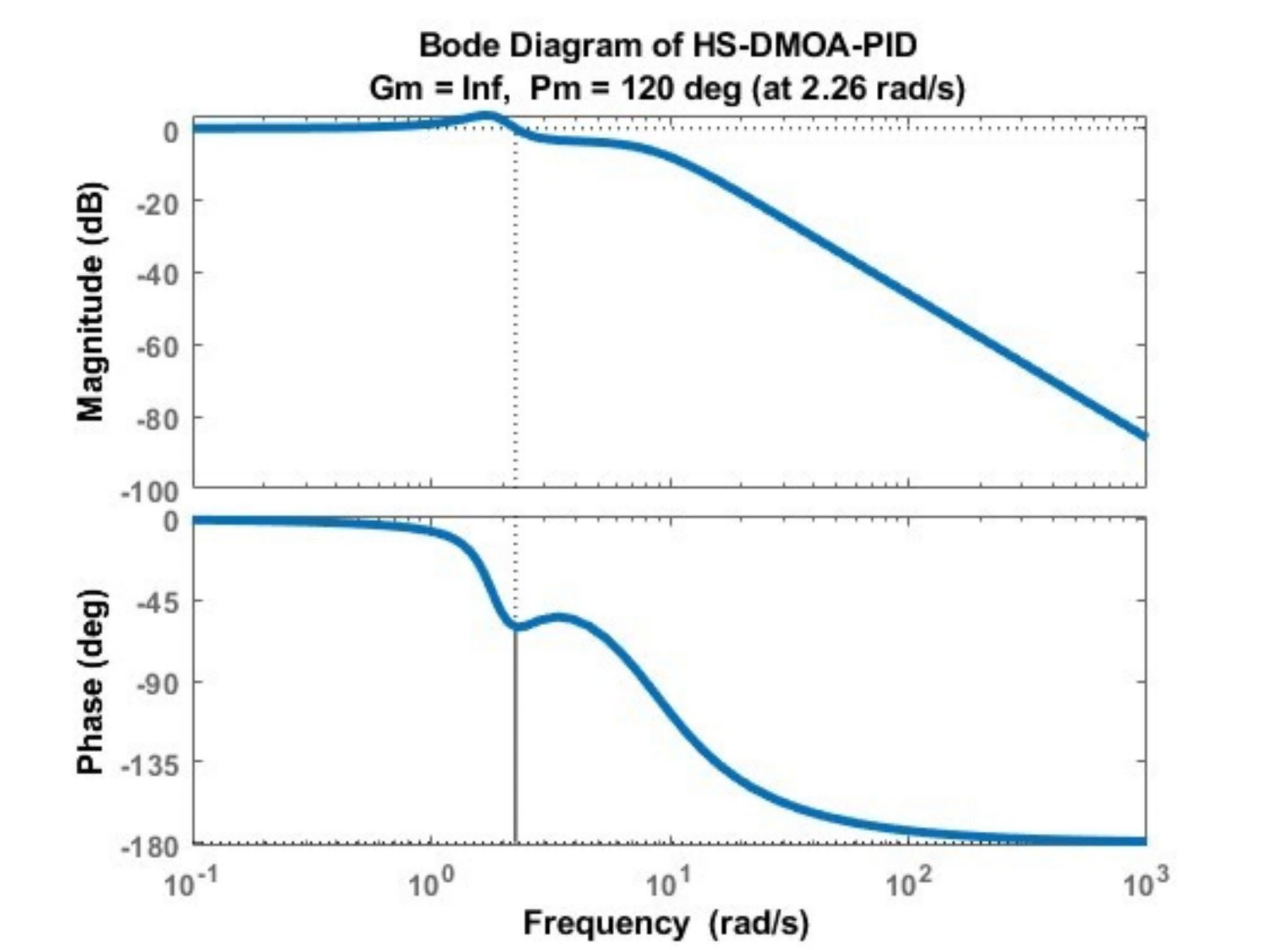
Bode Diagram of HS-DMOA-PID controller.

The Bode diagram of the HS-DMOA-PID is shown in Fig. 9.

It shows that the AVR system is stable. The PID HS-DMOA has a high level of frequency stability as shown by the bode diagram. A relatively higher phase margin indicates greater stability. The PID HS-DMOA shows a larger phase margin meaning that the system can tolerate more phase lag before reaching the critical stability boundary, while its narrow bandwidth reflects greater overall stability against variations in system parameters or disturbances.

Table 5 demonstrates the values of the phase margin, delay margin, and bandwidth of each method incorporating the PID controller.

**Table 5:** Optimization techniques with PID controller poles and damping ratios.

Optimization method	Phase Margin (degrees)	Peak Margin (dB)	Delay margin (degrees)	Bandwidth (Hertz)
HS-DMOA-PID	166.9364	1.5382	1.6706	8.603
HSA-PID^6^	58.3	3.54	0.0525	22.1
LUS-PID^6^	59.6	3.41	0.0553	21.9
TLBO-PID^6^	59.5	3.42	0.0553	22

Numerically speaking, the PID HS-DMOA has: 38%, 37%, and 39% more phase margin, 104.7%, 104.1%, and 111.9% less peak margin, and 172%, 171%, and 173% less bandwidth than TLBO, LUS, and HSA, respectively – while maintaining a relatively high delay margin.

Due to the apparent superiority of the HS-DMOA-PID on other alternative PID-based optimization techniques, we decided to take it a step further and compare it with both its PIDA counterpart and other PIDA-based techniques.

### PIDA time response analysis

**Fig. 10:**
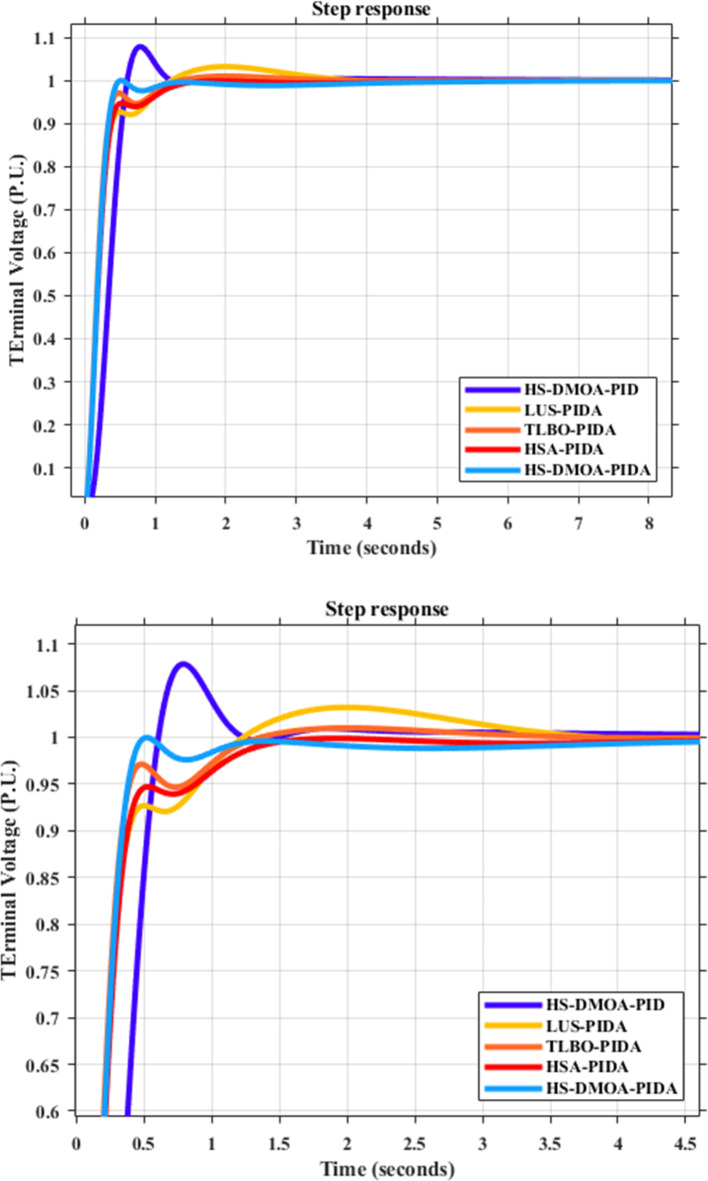
Output voltage of AVR system using HS-DMOA-PIDA controller V.S. other optimization techniques.

Figure 10 below showcases the performance of the system’s output voltage when employing the optimization technique PIDA HS-DMOA under a step disturbance.

Additionally, Table 6 presents the responses of various other optimization techniques. The Table highlights the adaptability of the system integrating the PIDA HS-DMOA optimization technique.

**Table 6:** Optimization techniques time indices response using PID controller.

Optimization method	*K*_*a*_	*K*_*d*_	*K*_*p*_	*K*_*i*_	α	*β*	Maximum overshoot	Rise time (sec.)	Settling time (sec.)
HS-DMOA-PIDA	129.207	527.119	905.561	356.811	508.905	890.241	1	0.273	0.932
HSA-PIDA[6]	143.256	520.545	845.675	360.278	565.45	970.784	1	0.313	1.16
LUS-PIDA[6]	149.096	486.846	783.442	447.048	552.567	919.971	1.0320	0.322	2.73
TLBO-PIDA[6]	150	550	850	421.6	550	900	1.002	0.273	1.06
HS-DMOA-PID [6]	N/A	0.2009	1.0040	0.3347	N/A	N/A	1.08	0.3	1.1

Numerically speaking, the HS-DMOA-PIDA obtained 13.7%, 192%, and 24% less settling time and, 17%, and 14% than TLBO-PIDA, LUS-PIDA, respectively with same overshoot as HSA-PIDA but with lower oscillation.

### PIDA root locus

**Fig. 11:**
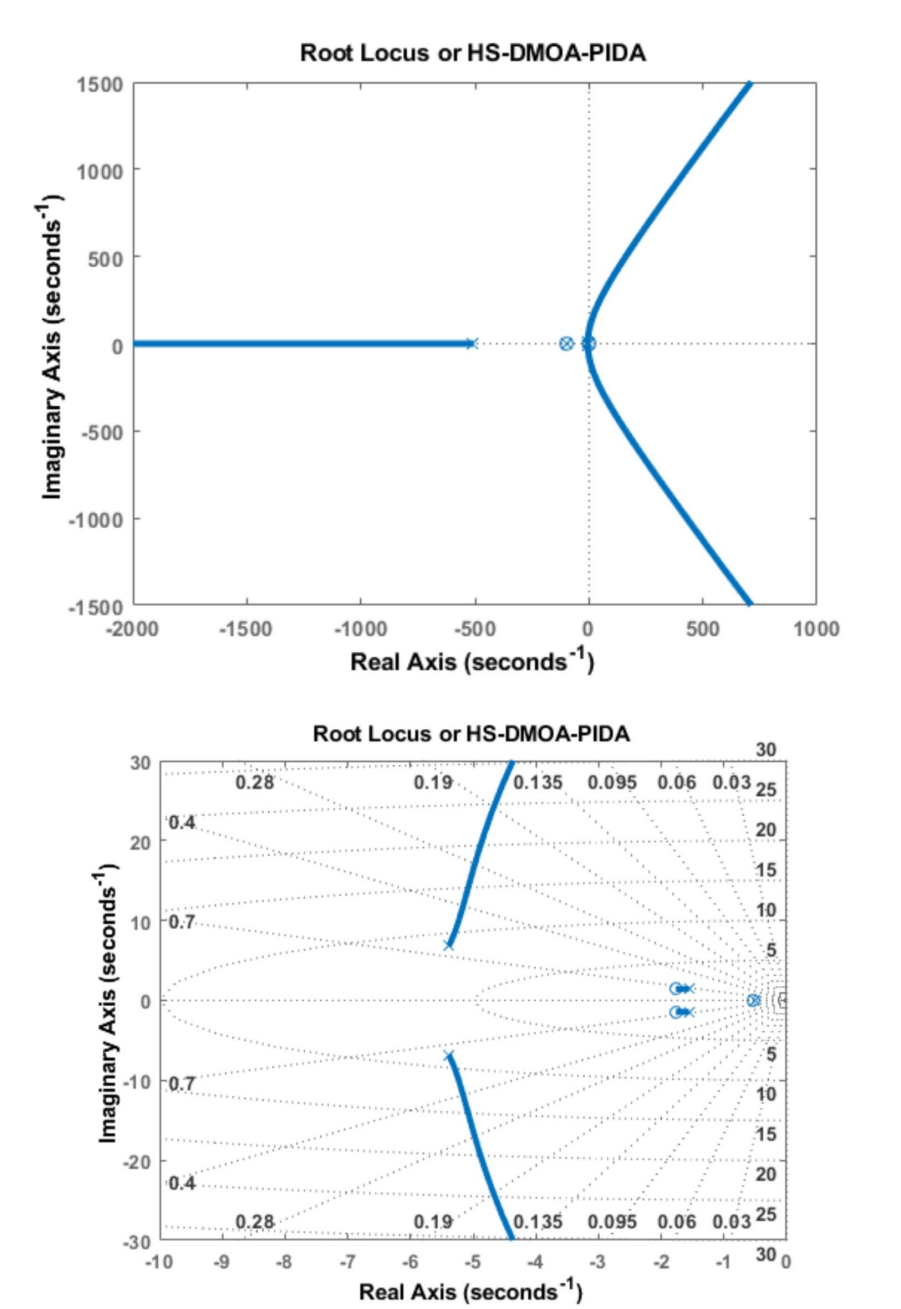
Root locus diagram of HS-DMOA-PIDA controller.

Figure 11 depicts the root locus diagrams of HS-DMOA applied to the AVR system alongside a PIDA controller. These diagrams are generated for the transfer functions outlined in Eq. (10).

Notably, the root locus analysis confirms the stability of the HS-DMOA-PIDA controller, evident from all roots residing on the negative side of the Real Axis in the S-plane.

Table 7 demonstrates how the eigenvalues and damping ratio are retrieved from the root locus diagram of each optimization method.

**Table 7:** Optimization techniques with PID controller poles and damping ratios.

HS-DMOA-PIDA	Pole	-550	-100	-1.01	-5.69 + 7.38i	-5.69–7.38i	-0.94 + 1.19i	-0.94–1.19i
	Damping ratio	1	1	1	0.6106	0.6106	0.6228	0.6228
LUS-PIDA^6^	Pole	-551	-101	-1.01	-5.69 + 7.38i	-5.69–7.38i	-0.95 + 1.19i	-0.95–1.19i
	Damping ratio	1	1	1	0.611	0.611	0.623	0.623
HAS-PIDA^6^	Pole	-564	-101	-0.596	-5.59 + 6.97i	-5.59–6.97i	-1.31 + 1.27i	-1.31–1.27i
	Damping ratio	1	1	1	0.625	0.625	0.718	0.718
TLBO-PIDA^6^	Pole	-548	-101	-0.855	-5.47 + 7.29i	-5.47–7.29i	-1.23 + 1.08i	-1.23–1.08i
	Damping ratio	1	1	1	0.6	0.6	0.751	0.751

### PIDA bode diagram

**Fig. 12:**
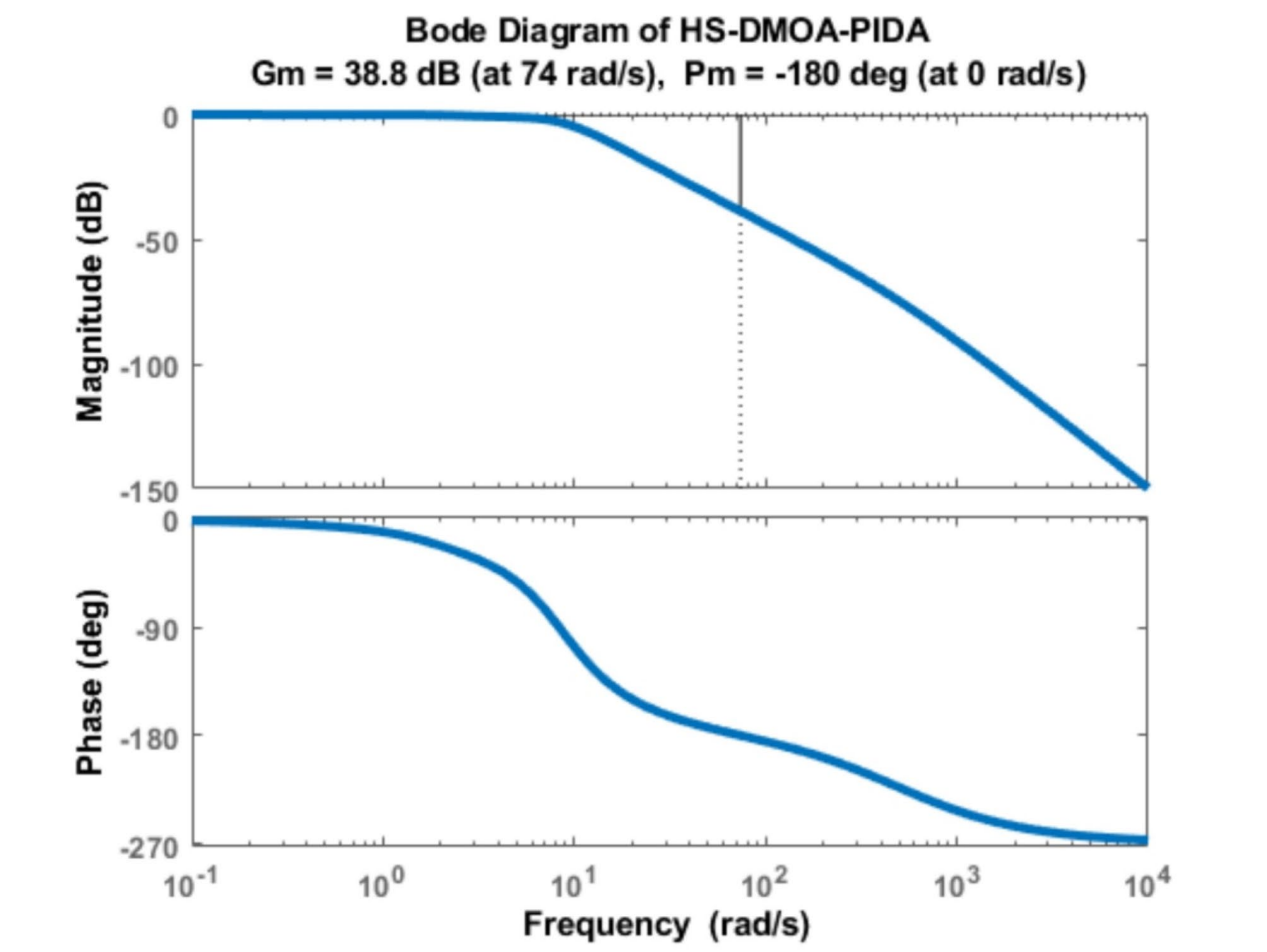
Bode Diagram of HS-DMOA-PIDA controller.

The Bode diagram of HS-DMOA PIDA is shown in Fig. 12. While, Table 7 demonstrates the values of the phase margin, delay margin, and bandwidth of each method incorporating the PIDA controller.

It shows that the AVR system is stable. The PIDA HS-DMOA has a high level of frequency stability as shown by the bode diagram. A relatively higher phase margin indicates greater stability. The PIDA HS-DMOA shows a larger phase margin meaning that the system can tolerate more phase lag before reaching the critical stability boundary.

**Table 8:** Optimization techniques frequency indices response using PID controller.

Optimization method	Peak Gain (dB)	Phase Margin (degrees)	Delay Margin (degrees)	Bandwidth (Hertz)
HS-DMOA-PIDA	0	180	Inf	8.3558
HSA-PIDA[6]	0	180	Inf	7.8612
LUS-PIDA [6]	0.15937	162.4758	2.42	8.1334
TLBO-PIDA [6]	0	180	Inf	8.7835
HS-DMOA-PID	96	1.67	0.2735	8.064

As shown in Table 8, the HS-DMOA-PID scored a record-high distinction with a 188%, 166%, 158%, and 174% less bandwidth compared to TLBO-PIDA, LUS-PIDA, HAS-PIDA, HS-DMOA-PIDA, respectively, where the HS-DMOA-PIDA had similar outputs compared to other PIDA-based techniques in terms of phase, peak, and delay margins and bandwidth.

### Dynamic disturbance

**Fig. 13:**
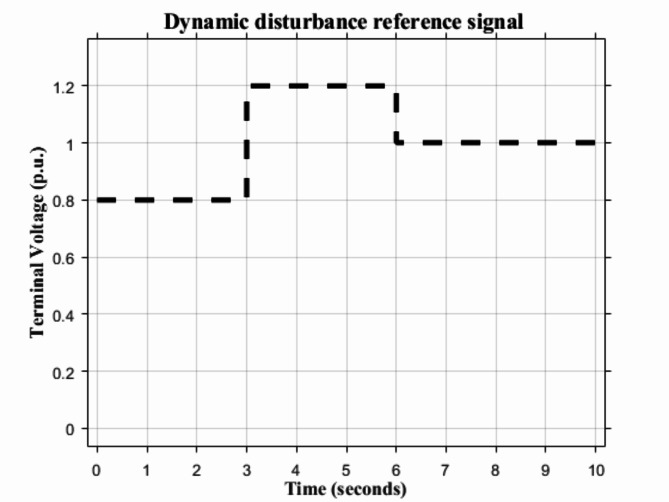
Dynamic disturbance signal with three p.u. levels over a 10 s period.

**Fig. 14:**
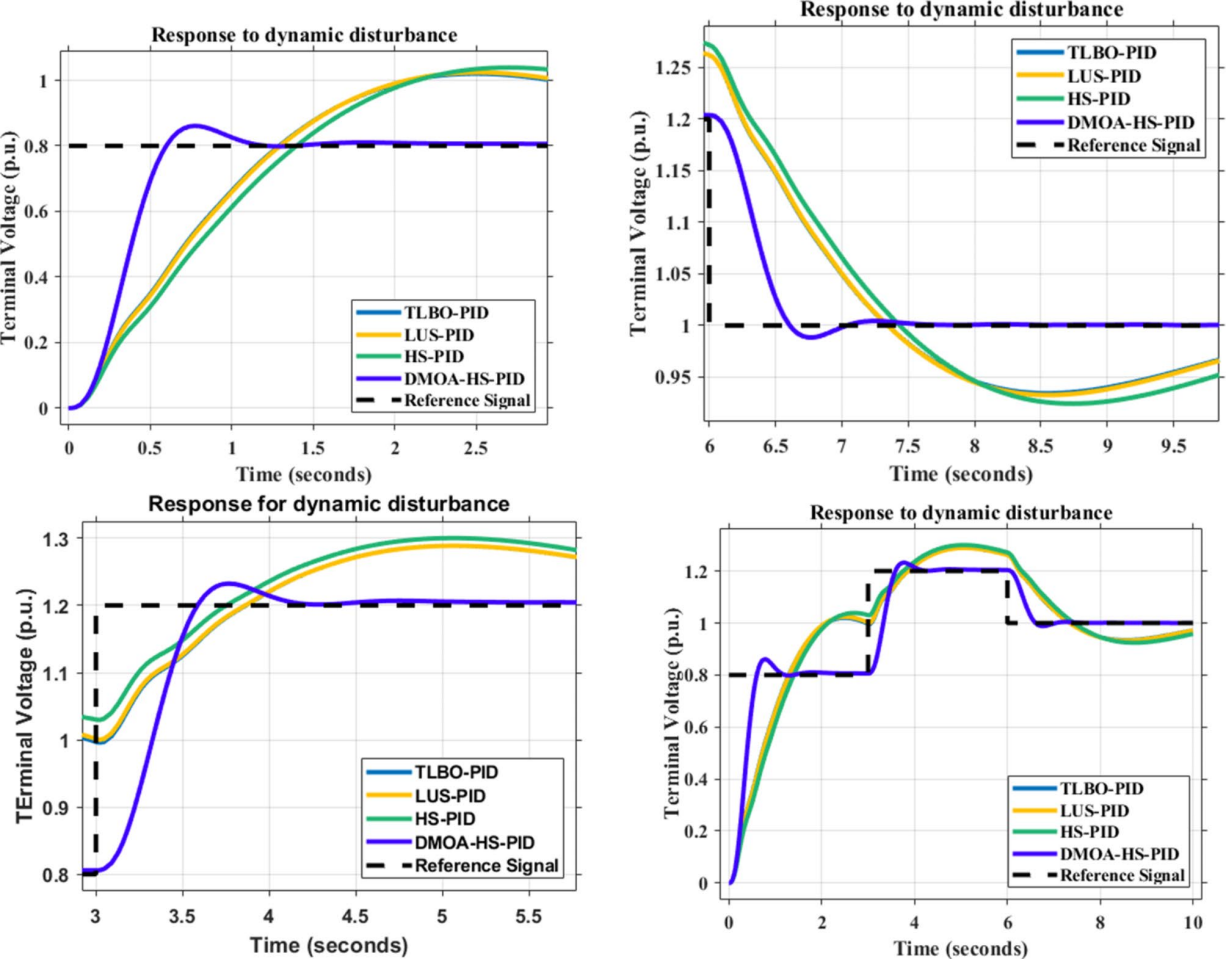
Time response for different PID-based optimization methods due to dynamic disturbance response.

**Fig. 15:**
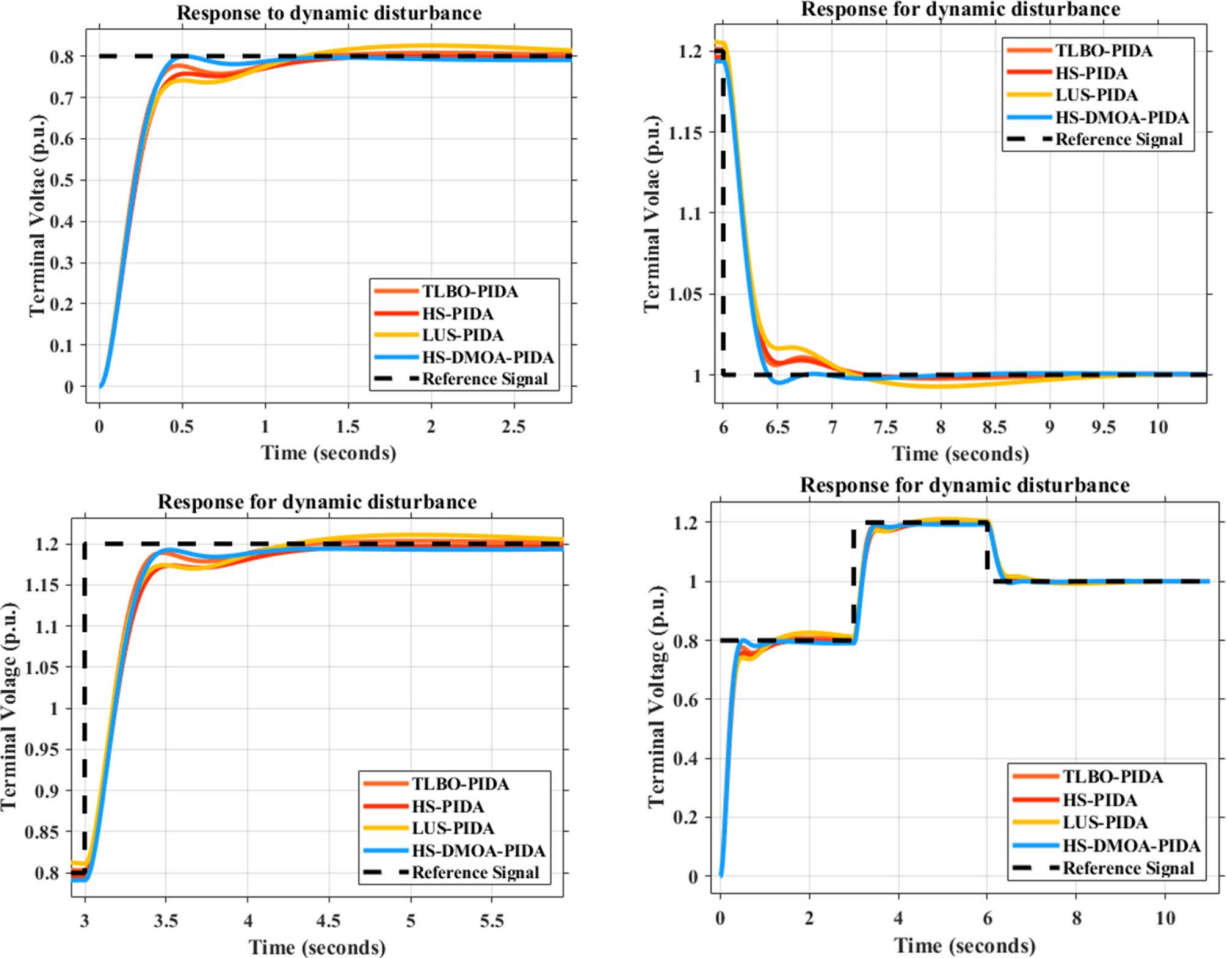
Time response for different PIDA-based optimization methods due to dynamic disturbance response.

The dynamic disturbance shown in Fig. 13 is applied to the proposed HS-DOMA PID and PIDA based and the other methods in the literature. Figures 14 and 15 demonstrate how the proposed HS-DMOA PID and PIDA based controllers follow the reference signal in the time domain in comparison with other optimization techniques. Tables 9 and 10 exhibit the time domain parameter of maximum overshoot for different optimization techniques with respect to the suggested HS-DMOA PID and PIDA based controllers.

**Table 9:** Maximum percentage overshoot of PID-based optimization methods under dynamic disturbance.

PID-based Optimization method	0.8 step reference (*p*.u.)	1.2 step reference (*p*.u.)	1 step reference (*p*.u.)
**HS-DMOA**	0.861	1.233	0.988
**HS**	1.038	1.300	0.928
**LUS**	1.024	1.289	0.937
**TLBO**	1.020	1.289	0.938

**Table 10:** Maximum percentage overshoot of PIDA-based optimization methods under dynamic disturbance.

PIDA-based Optimization method	0.8 step reference (*p*.u.)	1.2 step reference (*p*.u.)	1 step reference (*p*.u.)
**HS-DMOA**	0.800	1.193	0.995
**HS**	0.758	1.174	1.006
**LUS**	0.741	1.174	1.016
**TLBO**	0.777	1.189	1.007

At the 0.8 p.u. reference signal, the HS-DMOA PID and PIDA based surpassed in overshoot HS, LUS, and TLBO performance by 20.55%,18.93%, and 18.46% and 5.25%,7.37%, and 2.87%, respectively. While at the 1.2 p.u. reference signal, the HS-DMOA PID and PIDA based surpassed in overshoot HS, LUS, and TLBO performance by 5.43%,4.54%, and 4.54% and 1.59%, 1.59%, and 0.33%, respectively. At the 1 p.u. reference signal, the HS-DMOA PID and PIDA based surpassed in overshoot HS, LUS, and TLBO performance by 6.07%, 5.16%, and 5.06% and 1.10%, 2.11%, and 1.20%, respectively. Based on the previous data and the observation from Figs. 14 and 15; Tables 9 and 10 it can be concluded that the proposed HS-DMOA PID and PIDA based technique outperformed other traditional optimization techniques in the sense that it follows the dynamic disturbance signal more efficiently and with the least maximum overshoot.

### Statistical analysis

**Fig. 16:**
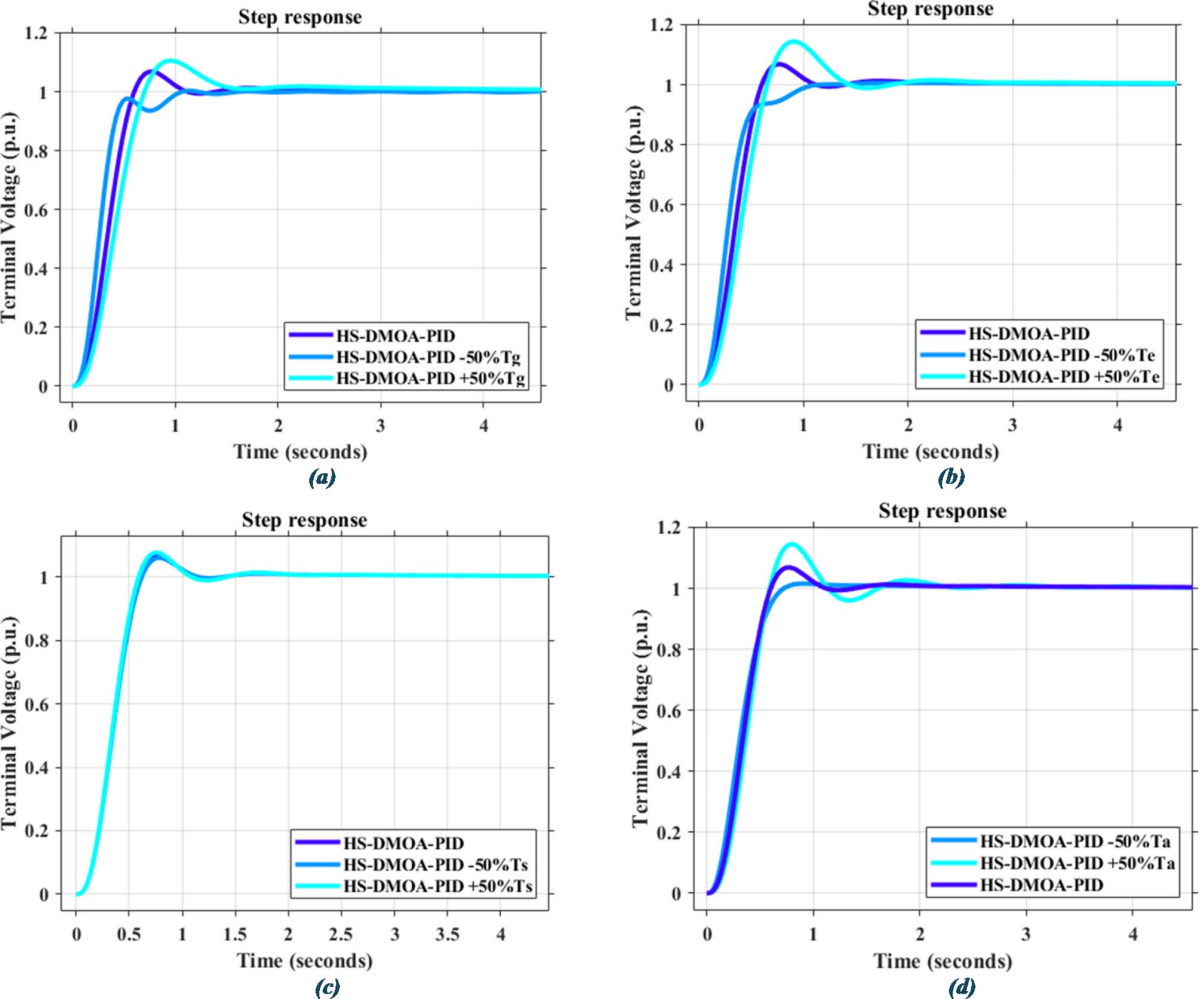
Time response for HS-DMOA-PID-based optimization method due to changes in time constants: (**a**) Tg (**b**)Te (**c**)Ts (**d**)Ta.

**Fig. 17:**
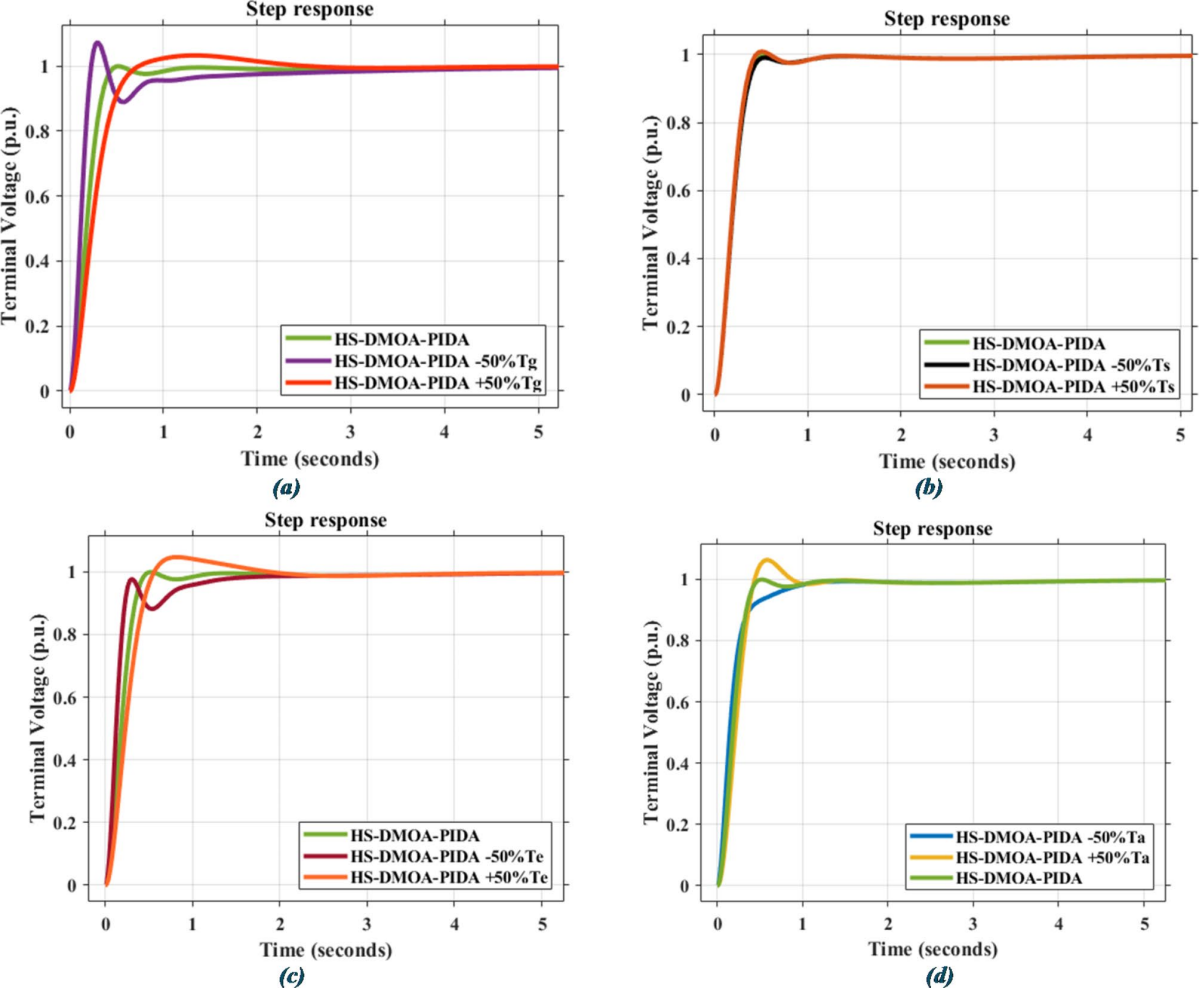
Time response for HS-DMOA-PIDA-based optimization method due to changes in time constants: (**a**) Tg (**b**)Te (**c**)Ts (**d**)Ta.

Below you can find how the PID-based AVR system responds to different disturbances due to ± 50% changes in time constants, T_a_, T_g_, T_e_, and T_s_, as shown in Fig. 16; Table 11, while Fig. 17; Table 12 correspond to PIDA-based response to the same disturbances.

**Table 11:** Power indices of PID-based AVR system after changes in different time constants.

Time constant	Percentage of change	Maximum overshoot (*p*.u.)	Rise time (seconds)	Settling time (seconds)
$\:{T}_{a}$	–50%	1.015	0.407	0.660
	+ 50%	1.143	0.357	2.036
$\:{T}_{e}$	–50%	1.006	0.361	0.954
	+ 50%	1.144	0.404	1.339
$\:{T}_{g}$	–50%	1.004	0.293	0.960
	+ 50%	1.106	0.425	1.454
$\:{T}_{s}$	–50%	1.061	0.367	1.017
	+ 50%	1.077	0.357	0.999

**Table 12:** Power indices of PIDA-based AVR system after changes in different time constants.

Time constant	Percentage of change	Maximum overshoot (*p*.u.)	Rise time (seconds)	Settling time (seconds)
$\:{T}_{a}$	–50%	0.95	0.32	1.00
	+ 50%	1.06	0.29	0.80
$\:{T}_{e}$	–50%	0.98	0.18	1.49
	+ 50%	1.05	0.36	1.40
$\:{T}_{g}$	–50%	1.07	0.14	2.50
	+ 50%	1.03	0.42	1.91
$\:{T}_{s}$	–50%	0.99	0.27	0.93
	+ 50%	1.00	0.27	0.93

As shown In Figs. 16 and 17; Tables 11 and 12, the proposed hybrid HS-DMOA PID and PIDA based show high stability in response to fluctuations under several time constant disturbances, while also not deviating far away from the original undisturbed response of the system.

## Discussion

The AVR model is utilized with a step response of one Per Unit (p.u.). Once again, the HS-DMOA-PIDA demonstrated reduced sensitivity to disturbances, shorter settling time, and enhanced overall system stability. Similarly, compared to alternative techniques like HS, LUS, and TLBO, HS-DMOA-PIDA exhibits superior stability and response to disturbance indicators, displaying lower maximum overshoot, settling time, and rise time.

## Validation through benchmark functions

In addition to the application superiority of the proposed HS-DMOA, this section proved its superiority through comparison with HS, DMOA, and one of the classical optimization methods (LUS) on the Unimodal benchmark functions (F1-F7) given in^62^, as shown in Table 13.

**Table 13:** Statistical results of optimization methods on different benchmark functions.

Function	Global optima		HS	DMOA	HS-DMOA	LUS
F1	0	Max	9.88E + 02	0	0	9.90E-07
		Min	2.91E + 02	0	0	5.55E-07
		Avg	5.88E + 02	0	0	8.78E-07
		Std	2.13E + 02	0	0	1.34E-07
F2	0	Max	0	0	0	1.37E + 00
		Min	0	0	0	5.54E-02
		Avg	0	0	0	5.70E-01
		Std	0	0	0	4.72E-01
F3	0	Max	6.38E + 03	2.57E-04	3.09E-05	1.18E + 01
		Min	3.11E + 03	3.36E-05	1.11E-06	4.50E-01
		Avg	4.82E + 03	1.12E-04	9.25E-06	4.87E + 00
		Std	1.25E + 03	8.05E-05	8.92E-06	4.49E + 00
F4	0	Max	5.40E + 01	1.20E + 01	2.22E-02	8.42E-01
		Min	1.46E-08	2.90E-07	1.72E-09	1.83E-03
		Avg	1.78E + 01	1.20E + 00	2.22E-03	3.35E-01
		Std	1.51E + 01	3.80E + 00	7.04E-03	3.22E-01
F5	0	Max	4.50E + 05	1.62E + 01	6.23E + 00	8.97E + 00
		Min	2.86E + 03	1.86E + 00	1.31E-01	4.34E + 00
		Avg	1.28E + 05	5.79E + 00	2.84E + 00	6.63E + 00
		Std	1.27E + 05	3.81E + 00	2.26E + 00	1.55E + 00
F6	0	Max	7.70E + 02	0	0	9.92E-07
		Min	2.66E + 02	0	0	6.48E-07
		Avg	5.58E + 02	0	0	8.70E-07
		Std	1.52E + 02	0	0	1.04E-07
F7	0	Max	2.95E-01	6.09E-03	4.67E-03	5.42E-01
		Min	5.75E-02	2.46E-03	2.35E-03	2.63E-02
		Avg	1.69E-01	4.07E-03	3.39E-03	2.23E-01
		Std	8.12E-02	1.31E-03	7.51E-04	1.64E-01

It is clear that, the proposed HS-DMOA method outperformed HS, DMOA, and TLBO in F3, F4, F5, and F7, in terms of reaching global optima with better maximum, minimum, average, and standard deviation values. In addition, the proposed HS-DMOA has slightly the same performance as DMOA in F1 and F6. Also, it has the same performance as DMOA and HS in F2. Moreover, showing relatively low standard deviation values than the other techniques which indicates its stability.

## Conclusion

Enhancement of AVR system performance is carried out in this paper by using a novel hybrid technique between the Harmony Search (HS) and Dwarf Mongoose Optimization (DMO) algorithms to optimize the PID and PIDA parameters. The proposed PID HS-DMOA-PID-based has better time performance indices, where the proposed PID-HS-DMOA has settling time less than the PID-based HS by 636.36%, LUS by 590.9%, TLBO by 581.81%, SMA by 272.72%, RSA by 245.45%, and L-RSAM by 272.72%. The proposed PID HS-DMOA has lower overshoot than PID-based HS by 20.37%, LUS by 18.5%, TLBO by 18.5%, SMA by 2.77%, RSA by 5.55%, and L-RSAM by 2.77%. Finally, it had less rise time from PID-based TLBO by 213.33%, LUS by 216.6%, HS by 243.33%, SMA by 90%, RSA by 83.33%, and L-RSANM by 86.66%. With respect to the frequency domain, the proposed PID HS-DMOA technique has a better phase margin than PID-based HS by 39%, LUS by 37%, and TLBO by 38%. Additionally, the proposed PID HS-DMOA has a better peak margin than PID-based HS by 111.9%, LUS by 104.1%, and TLBO by 104.7%. Furthermore, PIDA HS-DMOA based proved its superiority than the PIDA-based on HS, LUS, TLBO, and PID HS-DMOA based. The PIDA HS-DMOA settling time is lower than PIDA-based HS, LUS, and TLBO based by 24%, 192%, 13.7%, respectively. Also, the overshoot of the PIDA HS-DMOA is lower than PIDA-based HS, LUS, and PID HS-DMOA based by 14%, 17%, and 20%, respectively. In addition, the proposed PIDA HS-DMOA has almost the same frequency response as PIDA-based HS, TLBO, and PID HS-DMOA, and all of them have a better performance than LUS. Finally, a robustness test is carried out by using a dynamic disturbance, the HS-DMOA PID and PIDA based outperformed PID and PIDA based HS, LUS, and TLBO by 20.55%, 18.93%, and 18.46%, and 5.25%, 7.37%, and 2.87%, at 0.8 p.u. reference signal, respectively. The HS-DMOA PID and PIDA based outperformed PID & PIDA based HS, LUS, and TLBO by 5.43%, 4.54%, and 4.54% and 1.59%, 1.59%, and 0.33%, respectively, during the 1.2 p.u. reference signal. The HS-DMOA PID and PIDA based outperformed PID and PIDA based HS, LUS, and TLBO by 6.07%, 5.16%, and 5.06% and 1.10%, 2.11%, and 1.20%, respectively, during the 1 p.u. reference signal. Moreover, the system shows high reliability against different time constant changes.

## Future work and expandability

The authors think that the topic of this paper has a great potential to expand in terms of the applications on different types of controllers, including Adaptive Neuro-Fuzzy Inference System (ANFIS) Controller, Fractional Order PID (FOPID) Controller, and Fuzzy Logic Controller (FLC), and on larger and more complex systems like Power Generation Plants (thermal, hydroelectric, nuclear, and renewable energy power plants), smart grids, and high-speed rail network.

## Data Availability

The datasets used and/or analyzed during the current study are available from the corresponding author on reasonable request.
